# Optimal timing of cranioplasty post-decompressive craniectomy in traumatic brain injury: a systematic review, meta-analysis, and overview of ongoing trials

**DOI:** 10.1007/s00701-025-06759-2

**Published:** 2026-01-08

**Authors:** Ashviniy Thamilmaran, Shaan Patel, Shiva A. Nischal, Honey Panchal, Kush Kale, Pious D. Patel, Jack Jallo, James S. Harrop

**Affiliations:** 1 University College London Medical School, 74 Huntley St, London, WC1E 6DE UK; 2 Victor Horsley Department of Neurosurgery, National Hospital for Neurology and Neurosurgery, University College London Hospitals NHS Trust, Queen Square, London, WC1N 3BG UK; 3 Department of Physiology, Anatomy & Genetics, Medical Sciences Division, University of Oxford, Oxford, OX1 3PT UK; 4 Department of Neurological Surgery, Thomas Jefferson University Hospital, Philadelphia, PA 19107 USA

**Keywords:** Complications, Cranioplasty, Craniectomy, Decompressive craniectomy, Functional outcome, Traumatic brain injury

## Abstract

**Background:**

The optimal timing of cranioplasty (CP) following decompressive craniectomy (DC) for the management of traumatic brain injury (TBI) remains debated. Prior studies comparing early CP (EC) and late CP (LC) report conflicting outcomes, compounded by inconsistent timing thresholds and limited attention to effect modifiers such as implant material.

**Objective:**

To perform a systematic review and meta-analysis comparing outcomes of EC (≤ 90 days) versus LC (> 90 days) after DC for TBI, with particular evaluation of ultra-EC (< 35 days) and implant material.

**Methods:**

MEDLINE, Embase, and CENTRAL were electronically searched from inception to April 2025, supplemented by manual screening of references and grey literature. Randomised and observational studies comparing EC and LC in adult TBI patients were included. Primary outcomes of interest were overall complications, reoperation, and functional outcomes. Secondary outcomes included hydrocephalus, shunt dependence, extra-axial collections, infection, haematoma, bone resorption, seizures, mortality, and operative time. Risk of bias was assessed with ROBINS-I and RoB 2 tools, and certainty of evidence with GRADE. Pooled risk ratios (RRs) and mean differences (MDs) were calculated using random-effects meta-analysis.

**Results:**

Eighteen studies (*n* = 2226) were included. Overall complications did not differ between EC and LC, though autologous/allogenic EC carried higher risk (RR = 1.92; *P* = 0.02). Reoperation was significantly higher in mixed-materials EC cohorts (RR = 2.98; *P* = 0.02). No difference was observed in functional outcomes. Ultra-EC was associated with a lower risk of postoperative hydrocephalus (RR = 0.31; *P* = 0.005), while shunt dependence showed no significant difference. No significant differences were observed in extra-axial collections, infection, haematoma, bone resorption, seizures, or mortality. Operative time was shorter with EC (MD = -23.94 min; *P* = 0.0008), with the greatest reductions in ultra-EC (MD = -42.43 min; *P* < 0.00001). These findings are based largely on observational data with low-moderate certainty and should be interpreted cautiously.

**Conclusions:**

CP timing alone does not determine safety or efficacy, with risks varying substantially by implant material. Outcomes are critically modified by implant material and perioperative context. Ultra-EC may confer operative and physiological advantages without excess infection or mortality, particularly with synthetic implants, whereas early autologous or allogenic reimplantation carries higher risk of complications and reoperations. These findings argue for moving beyond a simplistic early-versus-late dichotomy and instead shifting towards material- and patient-specific strategies. Harmonised definitions and material-stratified prospective trials incorporating long-term functional outcomes are essential to establish evidence-based guidelines.

**Supplementary Information:**

The online version contains supplementary material available at 10.1007/s00701-025-06759-2.

## Introduction

Traumatic brain injury (TBI) is a major global health concern and the leading cause of injury-related death and disability, with an estimated 50–60 million people living with TBI worldwide [19]. Beyond the acute insult, TBI is associated with chronic cognitive and behavioural sequelae, generating a profound socioeconomic burden for patients, families, and healthcare systems [40]. Despite advances in critical care and neurotrauma management, mortality and morbidity from severe TBI remain high [40].

In severe TBI, secondary injury mechanisms such as cerebral oedema and uncontrolled intracranial hypertension drive poor outcomes. When intracranial pressure (ICP) remains refractory to maximal medical therapy, decompressive craniectomy (DC), or the surgical removal of part of the skull, is indicated to alleviate ICP and prevent secondary ischaemic injury. The landmark RESCUEicp trial demonstrated that DC significantly reduces six-month mortality in patients with refractory intracranial hypertension compared with medical management, but this benefit comes at the cost of increased risk of persistent vegetative state and severe disability [12]. These findings highlight the need to optimise the post-DC recovery pathway to improve long-term functional outcomes.

While DC is often necessary to prevent mortality, the creation of a large calvarial defect leaves the brain unprotected and physiologically compromised. Cranioplasty (CP) is therefore necessary once cerebral swelling has subsided to restore the protective barrier of the skull, either via implantation of a native bone flap from the patient (autologous), donor bone flap (allogenic), or a synthetic implant. Of note, physiological studies have demonstrated measurable improvements in cerebral blood flow and cerebrospinal fluid (CSF) dynamics documented on transcranial Doppler and perfusion imaging [36, 37] and small prospective cohorts reporting neurological recovery after early reconstruction [45]. However, no consensus regarding the optimal timing of CP has been reached, nor have standardised definitions for early CP (EC) versus late CP (LC) been determined. This variability contributes to significant heterogeneity in clinical practice.

Several clinical studies, systematic reviews, and meta-analyses have compared EC to LC, but findings and CP timing thresholds remain inconsistent. Zheng et al*.* [47] found EC (defined as ≤ 90 days) significantly reduced operative time and extra-axial fluid collections relative to later intervention, with no significant differences in overall complications or infection risk. Malcolm et al*.* [20] found that EC (defined as ≤ 3 months) was associated with greater postoperative neurological recovery compared to LC. De Cola et al*.* [6] also found better motor and cognitive recovery with EC (defined as ≤ 3 months). Palavani et al*.* [28] demonstrated EC (defined as ≤ 35 days) was associated with lower subdural effusion rates, shorter operative times, higher risk of hydrocephalus, though overall complication rate differences were inconclusive. Chasles et al*. *[2] conducted the most recent systematic review and found no consistent superiority of EC (defined as ≤ 90 days) versus LC (defined as > 90 days) for any outcomes. A recent multicentre prospective study from the CENTER-TBI and Net-QuRe collaborations found that 12-month functional outcomes were equivalent between EC (defined as ≤ 90 days) and LC (defined as > 90 days), but a significantly higher incidence of hydrocephalus with EC was noted [42]. Further study is necessary to determine whether the benefits of decreased hydrocephalus risk warrant delayed restoration to normal cranial physiology in LC, which is particularly relevant in the context of paediatric TBI patients who benefit from early neurorehabilitation [9, 48].

Collectively, previous literature suggests that EC may offer functional and technical advantages, though potentially at the expense of increased complication risk in certain patients. However, existing systematic reviews and meta-analyses share three major shortcomings that limit confidence in their findings. Firstly, heterogeneous definitions of “early” and “delayed” CP introduce potential misclassification bias. Secondly, most analyses relied heavily on retrospective studies with small sample sizes and insufficient adjustment for confounding variables. Finally, there has been limited exploration of potential effect modifiers including implant material and CSF shunt insertion.

To address these gaps, we conducted a systematic review and meta-analysis comparing EC (≤ 90 days) and LC (> 90 days) post-DC for TBI. Specifically, we evaluated postoperative complications, reoperation, and functional outcomes, while exploring the influence of ultra-EC (< 35 days) and implant material. The findings of this study aim to directly inform evidence-based recommendations and future trial design. Accordingly, the key question is not whether EC is universally superior to LC, but rather under which timing-material combinations CP can be performed safely and effectively.

## Materials and methods

This systematic review and meta-analysis followed the Preferred Reporting Items for Systematic Reviews and Meta-Analyses (PRISMA) guidelines [27] and the AMSTAR-2 methodological standards [35]. The AMSTAR-2 criteria were used to assess the quality of previously published systematic reviews in relation to this review. The study protocol was registered a priori on the Prospective Register of Systematic Reviews (PROSPERO ID: CRD420251032162).

### Search strategy and selection criteria

A comprehensive electronic search across three databases (PubMed, Embase, and CENTRAL) was conducted on 13th April 2025. The following search strategy was adapted for each database: (Cranioplasty[Mesh] OR cranioplasty OR "cranial reconstruction" OR "skull reconstruction" OR "skull repair" OR "bone flap replacement") AND ("Decompressive Craniectomy"[Mesh] OR "decompressive craniectomy" OR "decompression surgery") AND ("Brain Injuries, Traumatic"[Mesh] OR "traumatic brain injury" OR TBI OR "head injury" OR "head trauma") AND (timing OR "early cranioplasty" OR "late cranioplasty" OR "delayed cranioplasty" OR early OR delayed OR late) (Supplementary Table  1 A). The search was limited to English-only articles. The reference lists from all included studies and previous systematic reviews were manually searched to identify any additional relevant studies.

Eligibility criteria for this study included randomised or observational studies enrolling adult participants (≥ 18 years) comparing the use of EC (≤ 90 days) versus LC (> 90 days) in TBI patients and reporting at least one clinical outcome of interest. The following study designs were excluded: case reports, case series, review articles, conference abstracts, technical notes, cadaveric studies, studies with no outcome data, studies in non-English languages without translation. No minimum follow-up threshold was imposed a priori, consistent with prior reviews, to avoid excluding otherwise informative cohorts (however, follow-up is explicitly quantified and discussed in the results and limitations sections). Primary outcomes of interest were: (i) overall complications defined as the proportion of patients experiencing any postoperative complication; (ii) reoperation defined as a secondary surgery needed to treat a complication of the primary CP; (iii) functional outcome as per Glasgow Outcome Scale (GOS) at last follow up. Secondary outcomes of interest were: (i) hydrocephalus identified radiologically; (ii) hydrocephalus requiring CSF shunt insertion; (iii) extra-axial fluid collection defined as any new intracranial fluid accumulation outside brain parenchyma identified on postoperative imaging, regardless of need for intervention; (iv) postoperative infection defined as any local or systemic infection following CP; (v) haematoma defined as any clinically significant intracranial/extracranial blood collection post-CP confirmed by imaging or requiring intervention; (vi) bone resorption defined as radiological or clinical evidence of partial or complete loss of the autologous bone flap requiring monitoring or revision surgery; (vii) seizures defined as new-onset post-CP; (vii) mortality as defined as death either in-hospital or during follow-up; (viii) operative time measured in minutes.

Two authors (A.T. and S.P.) independently performed title-and-abstract screening of retrieved articles using Rayyan [26], with full texts subsequently assessed for inclusion. Any discrepancy was resolved by consensus discussion with a third reviewer (S.A.N.). Inter-rater reliability was calculated using Cohen’s kappa score (κ = 0.78), indicating almost perfect agreement between reviewers [16]. Where appropriate, subgroup analysis was conducted to investigate if there was a disproportionate impact on significance caused by: (i) ultra-EC (< 35 days) versus other (≥ 35 days); (ii) only autologous or allogenic versus only synthetic versus mixed bone flap material (defined as studies that did not use a single implant type exclusively).

We additionally searched unpublished randomised-controlled trials (RCTs) on ClinicalTrials.gov, WHO International Clinical Trials Registry Platform (ICTRP), International Standard Registered Clinical/soCial sTudy Number (ISRCTN), Australian New Zealand Clinical Trials Registry (ANZCTR), the Brazilian Registry of Clinical Trials (ReBEC), Chinese Clinical Trial Registry (ChiCTR), Korean the Clinical Research Information Service (CRIS), The Clinical Trials Registry—India (CTRI), Cuban Public Registry of Clinical Trials (RPCEC), EU Clinical Trials Register (EU-CTR), German Clinical Trials Register (DRKS), International Traditional Medicine Clinical Trial Registry (ITMCTR), Japan Registry for Clinical Trials (jRCT), Lebanese Clinical Trail Registry (LBCTR), Thai Clinical Trials Registry (TCTR), Pan African Clinical Trials Registry (PACTR), and Sri Lanka Trials Registry (SLCTR) (Supplementary Table 1B).

### Data analysis

Data from studies that fulfilled our inclusion criteria were extracted independently by two authors (A.T. and S.P.). Study demographics (author, date, country, design), total numbers of patients, patient demographics, and clinical outcomes of interest stratified by EC versus LC were extracted.

Two authors (A.T. and S.P.) independently evaluated certainty of evidence for each outcome (using the GRADE framework) [32] and risk of bias in included studies. Risk of bias in randomised studies was assessed using Cochrane’s Risk-of-Bias tool 2 (RoB 2) [39], while the Risk of Bias in Non-Randomised Studies of—Interventions tool (ROBINS-I) was used to assess non-randomised studies [38]. Disagreements were resolved through consensus discussion with a third reviewer (S.A.N.).

As per the pre-specified analysis plan, and in anticipation of moderate-to-high heterogeneity, a random effects meta-analysis of pooled raw data was employed using the Restricted Maximum-Likelihood (REML) model for each outcome with adequate data for quantitative synthesis. The results are presented in forest plots as mean difference (MD) for continuous outcomes and risk ratio (RR) for dichotomous outcomes. Corresponding 95% confidence intervals (CIs) were generated for each outcome. We assessed heterogeneity with Cochran’s Q test (*P* < 0.05 was considered statistically significant for heterogeneity) and I^2^ statistics (values > 25% was considered significant for heterogeneity). Significance level was set at α < 0.05 for this study.

Leave-one-out sensitivity analyses were conducted to investigate whether any particular study had a disproportionate contribution to results that were statistically significant. Publication bias was assessed using funnel plots in meta-analyses containing ≥ 10 studies for an outcome (power of the tests is too low to distinguish chance from real asymmetry if less than 10 studies). All analyses were performed using Review Manager (RevMan) Version 9.7.0 [31].

## Results

**Fig. 1:**
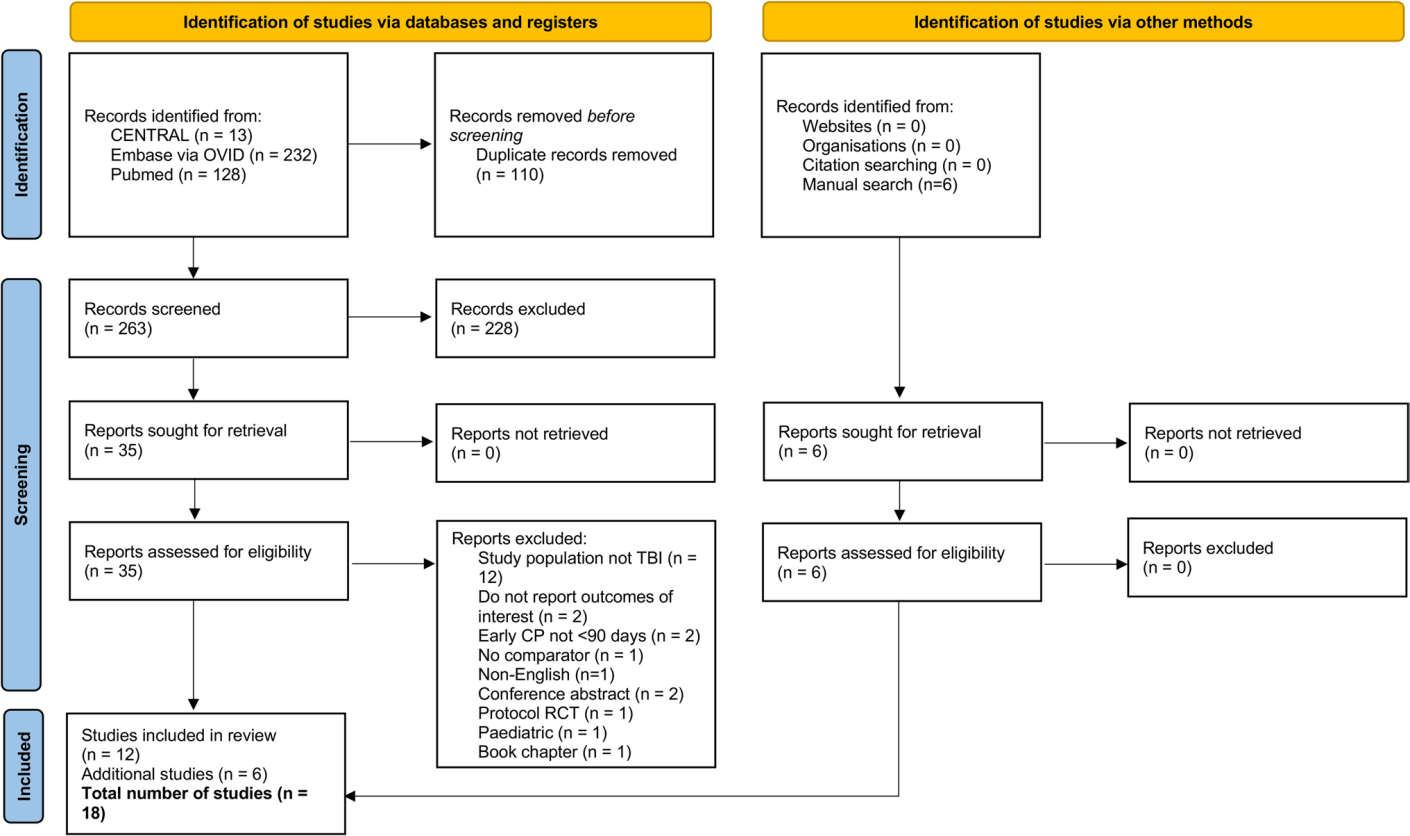
PRISMA  flow chart

A total of 379 studies were identified through database and grey literature searches, of which eighteen [3–5, 7, 14, 15, 23, 30, 33, 34, 36, 37, 41–45, 49] met inclusion criteria (Fig. 1). These comprised 13 retrospective observational studies [3–5, 7, 23, 30, 33, 34, 36, 41, 43–45] and 5 prospective observational studies [14, 15, 37, 42, 49], encompassing a cohort of 2,226 patients (819 EC; 1,407 LC). The studies originated from various countries including India [3, 15, 37, 43] (22.2%), South Korea [4, 5, 44] (16.7%), United States [7, 30, 34, 41] (22.2%), China [14, 36, 45, 49] (22.2%), Italy [23] (5.6%), Germany [33] (5.6%), and multi-national (multiple countries across Europe & Asia) [42] (5.6%) (Table 1). Follow-up reporting was heterogenous across studies (range ~ 0.5–30 months among those that reported), with several cohorts reporting ≤ 6 months (*n* = 10) and mutliple studies not reporting follow-up duration entirely (*n* = 6) (Table 2).

**Table 1:** Characteristics (study information, intervention information and funding sources) of included studies

First author (Year)	Country	Study design	Pathology	Sample Size *(n*)		Gender distribution (% male)		Timing definitions (in days)		Age, in years (mean ± SD or mean (range))		Funding
				**EC**	**LC**	**EC**	**LC**	**EC**	**LC**	**EC**	**LC**	
Chaturhvedi (2015)	India	RCS	TBI	20	54	NR	NR	≤ 90	> 90	NR	NR	No external funding reported
Cho (2011)	South Korea	RCS	TBI	15	21	86.7%	71.4%	≤ 42	> 42	51.40 ± 14.97	52.52 ± 15.52	No external funding reported
Chun and Yi (2011)	South Korea	Single centre RCS	TBI	30	15	56.7%	60%	≤ 30	> 90	49.33 (7–77)	49 (28–68)	No external funding reported
Eaton (2022)	USA	Single centre RCS	TBI	141	294	74.5%	75.5%	≤ 90	> 91	51.1 ± 18.3	51.4 ± 15.3	No external funding reported
Jiang and Wang (2020)	China	PCS	TBI	28	32	18.3%	43.3%	≤ 42	> 90	NR	NR	No external funding reported
Kumar (2018)	India	PCS	TBI	21	21	NR	NR	≤ 60	> 60	NR	NR	No external funding reported
Nasi (2018)	Italy	RCS	TBI	59	71	NR	NR	< 90	> 90	60.2 ± 8.9	59.2 ± 9.0	No external funding reported
Piedra (2014)	USA	Single centre RCS	TBI	78	79	73.1%	72.2%	< 84	≥ 84	27.2 ± 20.0	31.9 ± 17.1	No external funding reported
Schuss (2012)	Germany	Single centre RCS	TBI	54	226	54%	52%	≤ 60	> 60	39 ± 19	47 ± 15	No external funding reported
Sethi (2022)	USA	RCS	TBI	39	38	82.1%	68.4%	< 30	> 30	42.9 ± 16.0	35.9 ± 14.5	No external funding reported
Song (2014)	China	RCS	TBI	25	18	64%	55.6%	< 84	≥ 84	40.1 ± 16.52	44.1 ± 15.30	No external funding reported
Songara (2016)	India	Single centre PCS	TBI	6	10	83.3%	90%	< 90	> 90	34.5	38.7	No external funding reported
Tora (2021)	USA	Single centre RCS	TBI	51	91	NR	NR	≤ 90	> 90	NR	NR	No external funding reported
Vreeburg (2024)	Europe and Isreal	Multi centre PCS	TBI	73	100	70%	72%	≤ 90	> 90	43.7 ± 21.5	41.3 ± 23.7	European Union 7th Framework Programme for Research (Grant no. 602150). Hannelore Kohl Stiftung (Germany). OneMind (USA). Hersenstichting Nederland (Dutch Brain Foundation, Grant no. ps2014.06). Finnish Cultural Foundation. Finnish Medical Foundation. Orion Research Foundation. UCSF Bagan Family Foundation. NIHR Cambridge BRC, Royal College of Surgeons of England
Vyas and Singh (2021)	India	RCS	TBI	44	46	86.4%	91.3%	≤ 60	> 60	41.75 ± 13.4	45.61 ± 12.5	No external funding reported
Yang (2018)	South Korea	Multi centre RCS	TBI	15	144	NR	NR	< 35	≥ 35	NR	NR	No external funding reported
Zhang (2010)	China	RCS	TBI	23	47	65.2%	66%	< 90	> 90	38.7 ± 12.6	40.1 ± 13.5	No external funding reported
Zhu (2018)	China	PCS	TBI	97	100	66%	61%	≤ 60	> 60	47.19 ± 10.66	43.61 ± 9.89	Brain Clinic and Basic Research Team Program of the First People’s Hospital of Kunshan (Grant: KYC004)

*EC* Early Cranioplasty, *LC* Late Cranioplasty, *NR* Not Reported, *PCS* Prospective Cohort Study, *RCS* Retrospective Cohort Study, *TBI* Traumatic Brain Injury

**Table 2:** Characteristics (bone flap material type, outcomes and follow up) of included studies

First author (Year)	Bone flap material type used	Primary outcome measure	Secondary outcome measures	Length of follow-up (mean ± SD or mean (range), months)	
				**EC**	**LC**
Chaturhvedi (2015)	Mixed	Complication rate (infection, hematoma, dural tear, seizures, CSF leak, mortality)	Operative time; predictors of complications (timing < 3 months, age > 20 years, female sex)	NR	NR
Cho (2011)	Mixed	Functional recovery (Barthel Index of ADL at 1 month)	Complications (infection, subdural fluid collection, ventricular dilatation)	1	1
Chun and Yi (2011)	Synthetic	Operative time, dissection time, blood loss	Complications (infection, subdural fluid collection, dural tears, soft tissue injury)	6	6
Eaton (2022)	Mixed	Complication rate (infection, hematoma, hydrocephalus, seizures)	Comparison of complications by timing: early (≤ 90 d), intermediate (91–180 d), late (> 180 d), ultra-early (< 42 d)	NR	NR
Jiang and Wang (2020)	NR	Functional recovery (KPS, ZPS, psychological function, QoL)	Complications (infection, fluid accumulation, scalp necrosis, intracranial hematoma)	NR	NR
Kumar (2018)	Autologous	Cognitive recovery (MMSE); functional recovery (GOSE)	Complications (seizures, EDH, ICH)	6	6
Nasi (2018)	Allogenic	Development of PTH requiring surgery	Functional recovery (6-month GOS)	6	6
Piedra (2014)	Mixed	Complication rate (infection, hydrocephalus, hematoma, bone graft resorption)	Operative duration; cost implications	18.7 ± 21.5	29.7 ± 31.6
Schuss (2012)	Autologous	Complication rate (epidural/subdural hematoma, abscess, hygroma, CSF fistula, wound healing disturbance, bone flap dislocation)	NR	NR	NR
Sethi (2022)	Autologous	Complication rate (infection, hydrocephalus, return to OR, bone flap removal)	Operative time	NR	NR
Song (2014)	Synthetic	CBF changes after CP	Complications (subdural fluid, infection, bone flap resorption, hydrocephalus, hemorrhage, wound issues, CSF leaks)	> 3	> 3
Songara (2016)	Synthetic	Cognitive recovery (MMSE); functional recovery (GCS, GOS)	Physiological measures (CBF, CBV, MTT); complications (hydrocephalus, seizures, trauma)	1	1
Tora (2021)	Mixed	Complication rate (overall, reoperation, hydrocephalus, infection, wound dehiscence, bone flap resorption, ischemic stroke)	Subgroup analysis (trauma vs non-trauma); complication severity (Clavien-Dindo classification)	NR	NR
Vreeburg (2024)	NR	Functional recovery (GOSE at 12 months)	QOLIBRI score; complications (hydrocephalus, seizures); subgroup analyses; inter-centre variability	12	12
Vyas and Singh (2021)	Mixed	Operative time; overall complication rate	Complications (infection, hematoma, hydrocephalus, sunken brain, bone resorption, reoperation); hospital stay; cost	0.50 ± 0.16	0.60 ± 0.28
Yang (2018)	Autologous or allogenic (surgeon’s discretion)	Earliest safe CP timing (serial CT); functional recovery (GOS at 6 months); overall complication rate	Complications (SSF, ventriculomegaly, hydrocephalus, hygroma, infection); predictors of good outcome	6	6
Zhang (2010)	Synthetic	Functional recovery (ADL score at 3 months post-DC, 1-month post-CP); long-term KPS; overall complication rate	Complications (wound issues, fluid collection, infection, epilepsy, meningeal tear); effect of duraplasty on recovery	≥ 6	≥ 6
Zhu (2018)	Synthetic	Functional recovery (GOS at 6 months); salivation improvement; scalp incision healing	Subgroup analyses by GCS; complications (infection, titanium mesh exposure); predictors of better outcomes	6	6

*ADL* Activities of Daily Living, *CBF* Cerebral Blood Flow, *CBV* Cerebral Blood Volume; *CP* Cranioplasty, *DC* Decompressive Craniectomy, *EAC* Extra-Axial Collection, *EC* Early Cranioplasty, *EDH* Extradural Hematoma, *GCS* Glasgow Coma Scale, *GOS* Glasgow Outcome Scale, *GOSE* Glasgow Outcome Scale – Extended, *ICH* Intracerebral Haemorrhage, *KPS* Karnofsky Performance Scale, *LC* Late Cranioplasty, *MMSE* Mini-Mental State Examination, *MTT* Mean Transit Time, *NR* Not Reported, *PTH* Post-Traumatic Hydrocephalus, *QOLIBRI* Quality of Life After Brain Injury, *SSF* Sinking Skin Flap Syndrome, *ZPS* Zubrod Performance Scale

This review was deemed to be high quality as per the AMSTAR-2 criteria (Supplementary Table 2).

### Primary outcomes

**Fig. 2:**
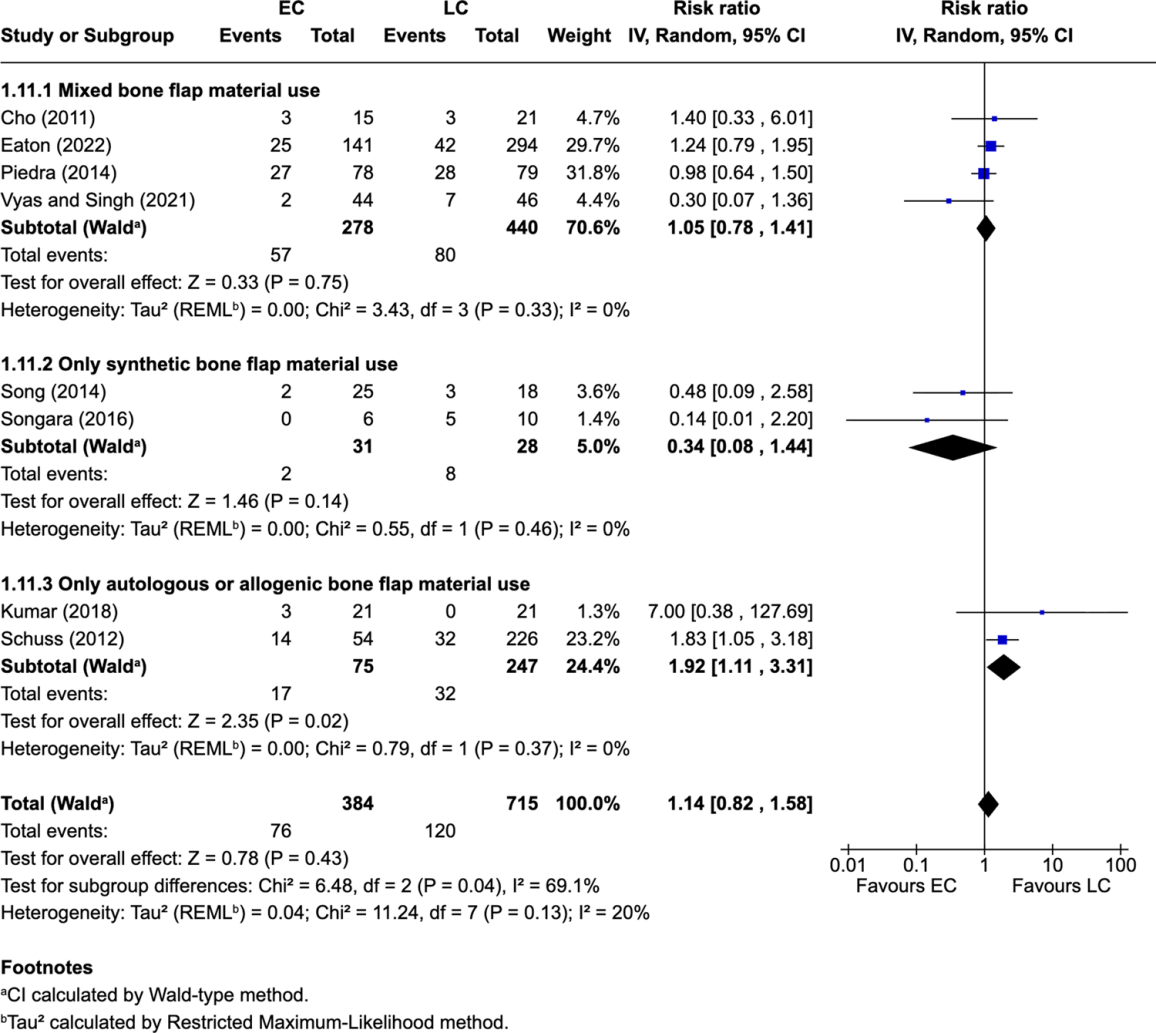
Overall complications with subgroup analysis

Eight studies [4, 7, 15, 30, 33, 36, 37, 43] (44.4%) reported overall complications. Complication events occurred in 19.8% of EC patients (76/384) and 16.8% of LC patients (120/715). The pooled RR was 1.14 (95% CI: 0.82–1.58; *P* = 0.43; I^2^ = 20%). In the autologous or allogenic bone flap material subgroup, complications occurred in 22.7% of EC patients (17/75) and 13.0% of LC patients (32/247), with a pooled RR of 1.92 (95% CI: 1.11–3.31; *P* = 0.02; I^2^ = 0%) in these limited cohorts (Fig. 2). Leave-one out sensitivity analysis was not conducted due to the subgroup comprising only two studies [15, 33].

**Fig. 3:**
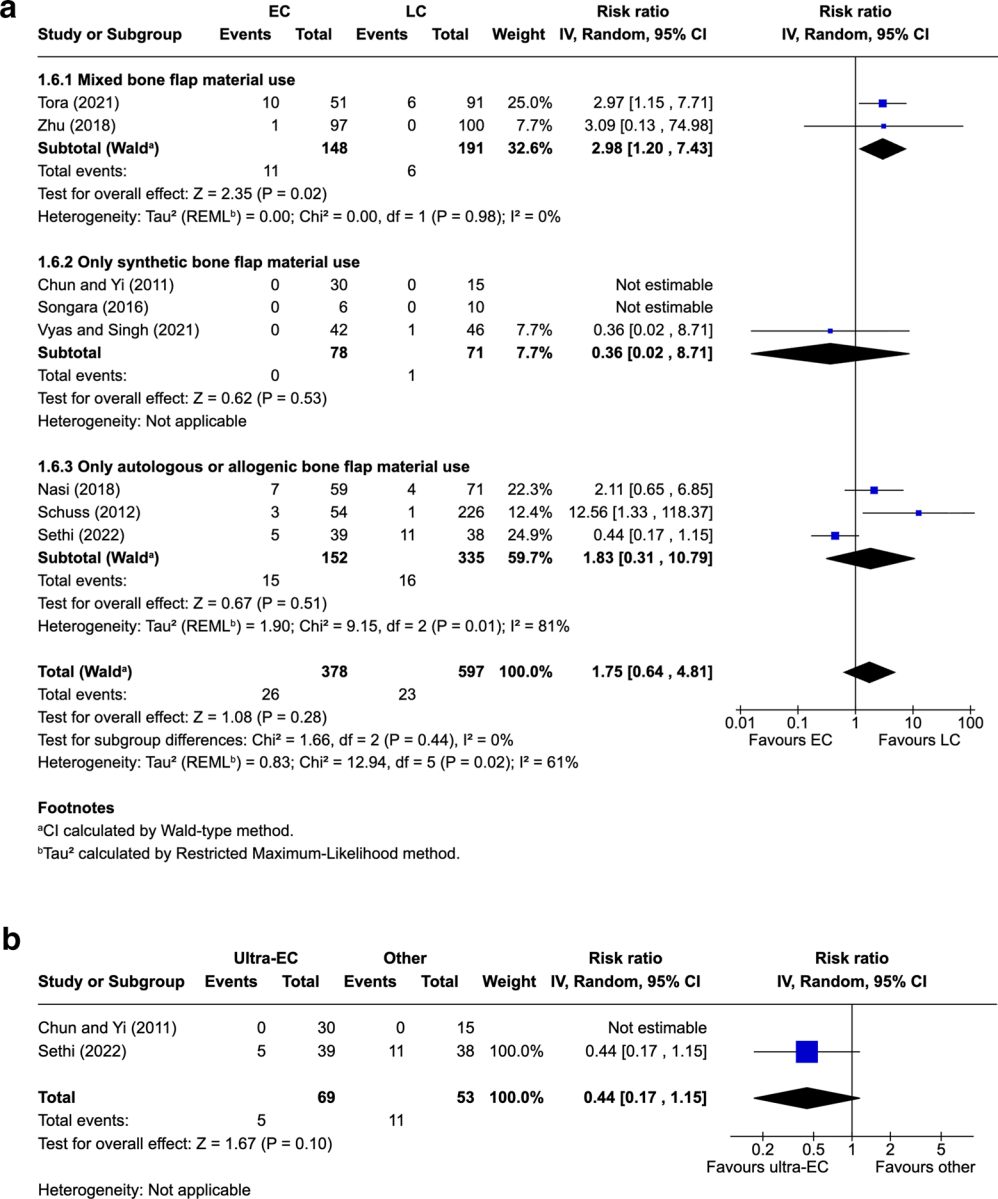
Reoperation with bone flap material subgroup analysis

Eight studies [5, 23, 33, 34, 37, 41, 43, 49] (44.4%) reported secondary reoperation following primary CP. Reoperation occurred in 6.9% of EC patients (26/378 patients) and 3.9% of LC patients (23/597 patients), with a pooled RR of 1.75 (95% CI: 0.64–4.81; *P* = 0.28; I^2^ = 61%). In the mixed bone flap material subgroup, reoperation occurred in 7.4% of EC patients (11/148) and 3.1% of LC patients (6/191), with a pooled RR of 2.98 (95% CI: 1.20–7.43; *P* = 0.02; I^2^ = 0%), although this subgroup consisted of only two studies [41, 49] (Fig. 3A). Leave-one out sensitivity analysis was not conducted,. In the ultra-EC subgroup, reoperation occurred in 7.2% of patients (5/69) compared with 20.8% of other patients (11/53), with a pooled RR of 0.44 (95% CI 0.17–1.15; *P* = 0.10) (Fig. 3B).

**Fig. 4:**
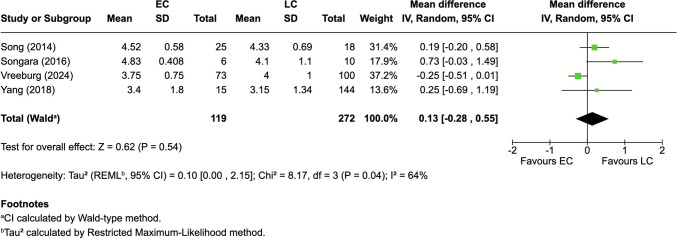
Functional outcomes scores

Four studies [36, 37, 42, 44] (22.2%) reported GOS at follow-up. Pooled analysis showed no significant difference between EC and LC (MD 0.13; 95% CI: −0.28–0.55; *P* = 0.54; I^2^ = 64%) (Fig. 4).

### CSF-related outcomes

Ten studies [4, 7, 23, 30, 34, 37, 41–44] (55.5%) reported postoperative hydrocephalus. Hydrocephalus occurred in 7.5% of EC patients (39/519) and 14.5% of LC patients (130/894), with a pooled RR of 0.90 (95% CI: 0.34–2.41; *P* = 0.83; I^2^ = 70%). In the mixed bone flap material subgroup, hydrocephalus occurred in 7.5% of EC patients (30/400) and 2.9% of LC patients (18/631), with a pooled RR of 2.63 (95% CI: 1.35–5.12; *P* = 0.005; I^2^ = 8%). Leave-one out sensitivity analysis showed that upon removal of Vreeburg et al*.* 2024 [42] from the subgroup analysis, the result was no longer statistically significant (*P* = 0.08). This subgroup is further limited by imprecision and residual confounding, and the certainty of evidence was rated low. In the autologous or allogenic bone flap material subgroup, hydrocephalus occurred in 8.0% of EC patients (9/113) and 43.5% of LC patients (110/253), with a pooled RR of 0.22 (95% CI: 0.10–0.45; *P* < 0.0001; I^2^ = 18%) (Supplementary Fig.  1 A). Leave-one out sensitivity showed that no single study had a disproportionate effect on statistical significance. In the ultra-EC subgroup, hydrocephalus occurred in 11.1% of patients (6/54) compared with 41.8% of other patients (76/182), with a pooled RR of 0.31 (95% CI: 0.14–0.71; *P* = 0.005; I^2^ = 0%) in these limited cohorts. Leave one-out analysis was not conducted due to the subgroup comprising only two studies [34, 44] (Supplementary Fig. 1B).

Four studies [23, 30, 41, 44] (22.2%) reported postoperative hydrocephalus requiring insertion of a CSF shunt. These events occurred in 7.4% of EC patients (15/203) and 17.1% of LC patients (66/385). The pooled RR was 0.81 (95% CI: 0.06–11.73; *P* = 0.88; I^2^ = 86%), with wide CIs reflecting low certainty and substantial heterogeneity (Supplementary Fig.  1 C).

Eleven studies [3–5, 14, 23, 33, 34, 36, 41, 44, 45] (61.1%) reported postoperative extra-axial fluid collections. These events occurred in 9.5% of EC patients (34/359) and 18.5% of LC patients (140/757). The pooled RR was 0.70 (95% CI: 0.38–1.30; *P* = 0.26; I^2^ = 50%). Subgroup analysis showed no significant effect when stratified into ultra-EC versus other subgroups, nor when stratified by bone flap material type (Supplementary Fig.  2 A and 2B).

### Other complications

Seventeen studies [3–5, 7, 14, 15, 23, 30, 33, 34, 36, 37, 41, 43–45, 49] (94.4%) reported postoperative infection. Infection events occurred in 5.8% of EC patients (43/746) and 6.0% of LC patients (78/1307). The pooled RR was 0.96 (95% CI: 0.63–1.46; *P* = 0.85; I^2^ = 10%). Subgroup analyses showed no significant effect when stratified by bone flap material (Supplementary Fig.  3 A) or timing of CP (Supplementary Fig. 3B).

Nine studies [5, 7, 14, 15, 30, 33, 37, 43, 44] (50.0%) reported postoperative haematoma. Haematoma events occurred in 5.1% of EC patients (21/415) and 7.2% of LC patients (62/867). The pooled RR was 1.02 (95% CI: 0.61–1.70; *p* = 0.95; I^2^ = 0%). Subgroup analysis showed no significant effect when organised into ultra-EC versus other subgroups nor when stratified by bone flap material type (Supplementary Fig.  4 A and 4B).

Four studies [30, 34, 41, 43] (22.2%) reported bone resorption. Resorption events occurred in 6.2% of EC patients (13/210) and 8.3% of LC patients (21/254). The pooled RR was 0.72 (95% CI: 0.38–1.35; *P* = 0.31; I^2^ = 0%) (Supplementary Fig. 5).

Six studies [3, 7, 15, 37, 42, 45] (33.3%) reported postoperative seizures. Seizure events occurred in 7.0% of EC patients (20/284) and 6.1% of LC patients (32/526), with a pooled RR of 1.15 (95% CI: 0.65–2.04; *P* = 0.63; I^2^ = 3%). Subgroup analysis showed no significant effect when organised into bone flap material subgroups (Supplementary Fig. 6).

### Mortality

Fourteen studies [3–5, 7, 15, 30, 33, 36, 37, 41–44, 49] (77.8%) reported mortality. Mortality occurred in 2.4% of EC patients (16/670) and 2.3% of LC patients (28/1219), with a pooled RR of 1.51 (95% CI: 0.80–2.85; *P* = 0.21; I^2^ = 0%) (Supplementary Fig.  7 A). Subgroup analysis showed no significant effect when organised into ultra-EC versus other subgroups nor when stratified by bone flap material type (Supplementary Fig. 7B).

### Operative efficiency

Five studies [5, 30, 33, 34, 43] (27.8%) reported operative time. Pooled analysis indicated shorter operative time in EC relative to LC (MD = −23.94 min; 95% CI: −37.94—−9.95; *P* = 0.0008; I^2^ = 88%). Leave-one out sensitivity analysis confirmed the stability of these findings (Supplementary Fig.  8 A). In the ultra-EC subgroup, MD was −42.43 min relative to the other subgroup (95% CI: −51.25—−33.61; *P* < 0.00001; I^2^ = 0%) (Supplementary Fig. 8B).

### Publication bias, risk of bias, and quality assessment

Funnel plot inspection demonstrated symmetry (six studies on either side of overall effect line), with all studies found beneath the 95% confidence interval pyramidal lines (Supplementary Fig. 9), suggesting low likelihood of publication bias. Egger’s regression test showed no evidence of small-study effects (*P* = 0.936). Of the 18 studies, 10 were judged low risk of bias and 8 moderate risk (Supplementary Table 3). Confounding was the most frequent source of bias. Certainty of evidence was assessed using the GRADE framework (Supplementary Table 4). Across the three primary outcomes, the certainty was judged to be moderate, and downgrading was due to risk of bias. For subgroup outcomes, the certainty of evidence was generally low or very low dure to imprecision, small study numbers, and residual confounding.

Quality assessment was conducted on five previous systematic reviews relevant to this topic. All were deemed to be critically low in quality as per the AMSTAR-2 criteria (Supplementary Table 5). Both Zheng et al. [47] and Malcolm et al. [20] critically failed to register their protocol a priori, assess publication bias or heterogeneity, and used the Newcastle–Ottawa Scale (NOS) for risk-of-bias assessment (deemed insufficient by AMSTAR-2). De Cola et al. [6] critically failed to register their protocol a priori, did not conduct any formal risk-of-bias assessment, and failed to integrate study-level quality into their interpretations. Palavani et al. [28] critically failed to register their protocol a priori, used the methodological items for non-randomized studies (MINORS) tool in the absence of domain-specific assessment (as per ROBINS-I and Cochrane’s RoB 2), omitted analyses of confounding and publication bias, and offered limited discussion of heterogeneity. Chasles et al. [2], the most recent review, inappropriately used the NOS for risk-of-bias assessment, and did not integrate bias assessments into conclusions.

### Overview of ongoing trials

A total of 155 articles were yielded through RCT database searches, with 4 relevant ongoing and recently completed trials investigating the optimal timing of CP after DC for TBI identified (Table 3). These included:

(i) Timing Impact of Early vs. Late Cranioplasty on Hemicraniectomy Outcomes (TIMELY) (NCT06632587): a prospective RCT currently recruiting patients in the USA comparing EC (defined as < 56 days) with LC (defined as > 90 days), using the modified Rankin Scale at 6 and 12 months to measure functional recovery as the primary outcome.
(ii) Effects of early skull repair with titanium mesh on cerebral blood flow and neurological recovery (NCT03222297): a Chinese RCT published in 2017, comparing EC (defined as ≤ 90 days) and LC (defined as > 182 days), reporting short-term improvements in cerebral perfusion [46].
(iii) Randomised Evaluation of Early vs Late Cranioplasty (REEL)—Cranioplasty Trial (ISRCTN14996072): a UK-based RCT completed in 2021 (currently unpublished) comparing EC (defined as ≤ 90 days) and LC (defined as > 90 days) – used extended GOS and quality of life metrics at 6 and 18 months.
(iv) Timing of Cranioplasty after Craniectomy in Patients with TBI (ChiCTR-TRC-12002571): a Chinese RCT comparing ultra-EC (defined as 28–42 days), EC (defined as 77–91 days), and LC (defined as 161–175 days), assessing functional, cognitive, and perfusion outcomes.

**Table 3:** Summary of ongoing trials and their characteristics. Published (Ya-Se et al. 2017)

	Timing Impact of Early vs. Late Cranioplasty on Hemicraniectomy Outcomes (TIMELY)	Effects of Early Skull Repair With Titanium Mesh on Cerebral Blood Flow and Neurological Recovery	Randomised Evaluation of Early vs Late Cranioplasty (REEL)—Cranioplasty Trial	Timing of Cranioplasty after Craniectomy in Patients with TBI
Source	ClinicalTrials.gov	ClinicalTrials.gov	ISRCTN	ICTRP
Study ID	NCT06632587	NCT03222297	ISRCTN14996072	ChiCTR-TRC-12002571
Country	USA	China	England	China
Number of centres	1	1	1	5
Sample size	Total (*n* = 44)	EC (*n* = 40) LC (*n* = 46)	Total (*n* = 50)	Ultra-EC (*n* = 50) EC (*n* = 50) LC (n = 50)
Study period	01/09/2024–01/09/2027	01/01/2013—31/01/2016	01/08/2019—31/08/2021	01/12/2012–31/12/2015
Status	Recruiting	Published (Ya-Se et al*.* 2017)	Complete	Recruiting
Most recent update	21/07/2025	N/A	22/03/2019	30/10/2015
Funding status	Thomas Jefferson University	Scientific Research Project for Health and Family Planning in Hubei Province (WJ2015MB308)	University of Cambridge Cambridge University Hospitals NHS Foundation Trust	Self-funded
Primary CP indication	TBI Stroke ICH	TBI	TBI Stroke (MCA infarct)	TBI
Population	Adults (≥ 18 years) Medically fit CCC A or B (at 4 weeks)	Adults	Adults (≥ 16 years)	Adults (18–60 years) Clinically stable No major comorbidities
Comparators	EC (< 8 weeks) LC (> 3 months)	EC (1–3 months) LC (6–12 months)	EC (< 3 months) LC (> 6 months)	Ultra-EC (4–6 weeks) EC (11–13 weeks) LC (23–25 weeks)
Primary outcomes	mRS (6 & 12 months)	CTP (3- & 10-days) Barthel index (30-days)	Extended GOS (6 months)	GCS Muscle strength MMSE Language ability GOS CTP
Secondary outcomes	Seizures (1 month) Hydrocephalus (1 month) Reoperation (1 month) Hospital readmission (1 month) Infection (12 months) Discharge disposition (12 months) Length of hospital stay (12 months)	Functional & neurological recovery comparisons between groups (CBF, CBV, TTP, Barthel)	QoL (EQ-5D, QOLIBRI) Psychological (NPI-Q) Surgical complications AEs Cost-effectiveness (EQ-5D)	Morphology (not specified) AEs (CP-related injury, wound infection, epilepsy, plate exposure, osteomyelitis, worsening neurological deficit)
Follow-up	6 months 2 months	3 days 10 days 30 days	18 months	NR
Randomisation protocol	NR	NR	Blocked randomisation (size of 4 or 6) with allocation ratio of 1:1	“Stata 7.0 program to generate random table (seed = 2010)”

*AE* Adverse Events, *CBF* Cerebral Blood Flow, *CBV* Cerebral Blood Volume, *CCC* Craniectomy Contour Class, *CSF* Cerebrospinal Fluid, *CTP* Computed Tomography Perfusion, *DC* Decompressive Craniectomy, *EC* Early Cranioplasty, *GCS* Glasgow Coma Scale, *GOS* Glasgow Outcome Scale, *ICH* Intracranial Haemorrhage, *LC* Late Cranioplasty, *MMSE* Mini-Mental State Examination, *mRS* Modified Rankin Scale, *NPI-Q* Neuropsychiatric Inventory Questionnaire, *N/A* Not Applicable, *NR* Not Reported, *QoL* Quality of Life, *TBI* Traumatic Brain Injury, *TTP* Time To Peak, *Ultra-EC* Ultra-Early Cranioplasty

## Discussion

This systematic review and meta-analysis examined the influence of CP timing after DC for TBI, with particular attention to ultra-EC (< 35 days) and the modifying role of implant material. Across 18 studies encompassing over 2200 patients, we found no significant differences between EC (≤ 90 days) and LC (> 90 days) in terms of overall complications, infections, mortality, or GOS-based functional recovery. However, subgroup analyses revealed clinically important distinctions. Ultra-EC was associated with shorter operative times and a lower reported incidence of hydrocephalus in limited data derived from only two retrospective cohorts, whereas autologous and allogenic implants used in EC procedures carried higher complication and reoperation risks. Collectively, these findings suggest that the impact of timing of CP is non-linear (challenging the traditional binary categorisation of “early” versus “late” CP) and cannot be considered in isolation from implant choice and perioperative context. This is particularly relevant as our subgroup analyses showed that early autologous or allogenic reimplantation carried higher complication and reoperation risks, whereas early reconstruction with synthetic implants appeared comparatively safer. These subgroup patterns, however, arise from predominantly observational data, with ROBINS-I assessments indicating moderate risk of bias and GRADE rating for the subgroup analyses typically low or very low.

### Complications and reoperation

The absence of a significant difference in overall complications between EC and LC aligns with previous meta-analyses, which consistently reported relative equipoise in pooled outcomes [2, 6, 28, 47]. Nevertheless, when stratified by implant type, EC with autologous or allogenic bone was associated with a nearly two-fold increase in complication risk (RR = 1.92). Similarly, reoperation risk was significantly higher when EC was performed in mixed-material cohorts (RR = 2.98), though this association disappeared in analyses restricted to synthetic or autologous/allogenic-only studies. Mechanistically, this excess risk may be driven by early reimplantation of biological flaps, in which immature scar formation, incomplete wound healing, and heightened local inflammation can predispose to dehiscence, infection, and resorption [22]. This has been observed clinically with Eaton et al*.* [7] reporting higher complication rates when autologous bone was reimplanted within 90 days, while Tora et al*.* [41] and Zhu et al. [49] found reoperation particularly elevated in mixed-material early cohorts. Early autologous reimplantation may also exacerbate aseptic bone flap resorption due to impaired revascularisation [17]. Osteopathological studies support this, showing cortical and cancellous disruption, osteoclastic activity, and residual fatty marrow impeding osteogenesis in resorbed flaps [10].

In contrast, synthetic materials such as titanium and polyetheretherketone (PEEK) offer structural stability and long-term durability. Unmodified PEEK is relatively biologically inert and does not reliably osteointegrate without surface modification, whereas titanium is highly biocompatible, corrosion-resistant, and provides structural support [18, 21]. The outcomes with these synthetic materials in this review were consistent regardless of timing, with very low risk of reoperation (0% in EC versus 1.4% in LC across synthetic only cohorts). These findings suggest that, where early restoration of cranial integrity is desirable, synthetic implants may minimise the risk of secondary surgeries. Conversely, when autologous bone is favoured, delaying CP until after the acute inflammatory phase (> 90 days) may reduce complication-driven revisions.

### Functional outcomes

Although our pooled GOS analysis did not demonstrate functional benefit of EC, smaller prospective cohorts have suggested otherwise. Kumar et al*.* [15] reported improved cognitive and functional outcomes with CP performed within 3 months, and Jiang & Wang [14] observed superior prognosis with “super-early” repair (< 30 days). Earlier physiological studies demonstrated improvements in cerebral blood flow and CSF dynamics after CP [34, 36], which may underpin these observations. More recently, Patel et al*.* [29] reported that ultra-EC (defined as ≤ 6 weeks) was not associated with increased complication rates and, in select patients, was linked with higher odds of long-term functional independence, although this association attenuated after adjustment for baseline characteristics, highlighting the importance of patient selection. Rehabilitation-focussed reviews have similarly suggested that earlier CP may facilitate motor recovery, though cognitive benefits are inconsistent across domains [6]. By contrast, a prior meta-analysis pooling change scores found greater overall neurological improvement with early CP [20]. Together these findings suggest that timing may influence rehabilitation potential in ways not captured by coarse ordinal scales.

### Hydrocephalus and CSF-related outcomes

Ultra-EC was associated with a lower reported incidence of postoperative hydrocephalus (RR = 0.31), although this finding remains based on a small number of observational cohorts and must be interpreted cautiously. This also contrasts with prior reports suggesting higher hydrocephalus rates with EC [28]. A plausible explanation is that earlier restoration of cranial integrity re-establishes CSF compliance and venous return, mitigating ventriculomegaly [11, 24]. Conversely, delayed CP may prolong abnormal CSF hydrodynamics and increase shunt dependence. This interpretation is supported by Honeybul and Ho et al*.* (2012) [10], who demonstrated that post-traumatic hydrocephalus following DC is largely driven by primary injury-related disruption of CSF drainage pathways rather than the craniectomy margin itself. Timely reconstruction may therefore facilitate earlier normalisation of CSF flow, which is also consistent with Yang et al*.* [44] who conducted a multicentre cohort study and found hydrocephalus risk varied depending on timing thresholds, with CP performed within 90 days associated with higher shunt dependence compared with late repair, and with Nasi et al. [23] who identified post-traumatic hydrocephalus risk to be strongly linked with DC-related factors such as subdural hygroma rather than CP timing alone.

In our analysis, EC was associated with numerically lower shunt-dependent hydrocephalus (7.4% in EC versus 17.1% in LC), though this did not reach statistical significance due to heterogeneity. Interestingly, trends diverged by material. In autologous/allogenic-only subgroups, EC reduced hydrocephalus risk, whereas in mixed-material cohorts, LC appeared protective. This suggests that material-specific inflammatory and integration processes modify CSF-related risks. However, these findings are derived from a small number of studies [34, 44] and require cautious interpretation. Notably, Patel et al*.* [29] found no significant difference in shunt dependence or hydrocephalus between ultra-EC (defined as ≤ 6 weeks), intermediate CP (defined as 6 weeks – 3 months), and LC (defined as > 3 months), further highlighting the variability across cohorts and the likely role of patient selection.

### Operative efficiency

EC was consistently associated with shorter operative duration (MD −23.94 min), with the largest reductions observed in the ultra-EC (−42.43 min). This likely reflects the technical advantage of reduced adhesion and fibrosis when reconstruction is performed prior to scar formation [1]. In the initial weeks post-DC, the dural surface remains relatively pliable, and overlying soft tissues have not yet undergone dense fibrotic remodelling, facilitating more straightforward surgical dissection. By contrast, delaying CP beyond 90 days allows for progressive scar maturation, dural thickening, and firm adhesion of the galea aponeurosis, temporalis muscle, and pericranial tissues to the underlying cortex and dura, often necessitating more extensive dissection. Clinically, this has been corroborated by Schuss et al*.* [33] who found significantly longer operative times and higher complication risk when CP was delayed beyond 3 months, and Chun & Yi [5] who similarly noted safer and more efficient dissection within the first month post-DC. In some cases, mature scar formation may also require adjunctive techniques such as sharp dissection or duraplasty, further increasing operative complexity [25]. Beyond technical considerations, operative efficiency carries pragmatic benefits including reduced anaesthetic burden, lower intraoperative bleeding, and decreased perioperative morbidity. In resource-limited settings, these efficiencies may influence surgical planning, though they must be weighed against the higher complication and reoperation risks associated with biological implants [13].

### Context within evolving practice

Over the last decade, the debate has shifted from a binary “early versus late” framing towards recognition that timing interacts with material, patient selection, and perioperative care. Our findings reinforce this evolution: EC with synthetic implants appears to be safe and efficient, whereas EC with autologous flaps is vulnerable to complications and resorption. The definitional heterogeneity of “early” (ranging from < 30 to < 90 days) further complicates cross-study synthesis [2, 6, 28, 47], echoing concerns raised in earlier correspondence that inconsistent thresholds hinder consensus [8]. Meanwhile, changes in practice, particularly the decline in cryopreserved autologous bone and the rise of custom synthetics, means that older studies may not reflect contemporary risks. The introduction of adjunctive radiographic tools such as the Craniectomy Contour Classification proposed by Patel et al*.* [29] may also aid patient selection, offering an objective framework to identify candidates suitable for ultra-early reconstruction. Future trial frameworks must align definitions, stratify by material, and adopt consistent outcome measures.

Furthermore, the reduced operative time with ultra-EC (MD 42 min shorter) is particularly relevant in the context of globally shifting priorities towards operative efficiency and value of care delivered per unit time. The shorter operative time, alongside the possibility that an earlier CP may result in long-term neurorehabilitation, may be a significant driver of policy change in the landscape.

### Implications for practice and research

Clinically, these results support a tailored approach. Ultra-EC may be beneficial where early restoration of cranial integrity is desired, particularly if synthetic implants are used. Conversely, when autologous bone is employed, delayed CP beyond 90 days may mitigate complication and reoperation risk. From a research standpoint, several priorities emerge. First, harmonised definitions of “early” and “late” CP are essential to reduce classification heterogeneity across studies. Second, future randomised and prospective multicentre studies must stratify by implant material to determine whether the safety advantages of synthetics observed here translate into clinical superiority. Third, outcome measures should extend beyond survival and crude GOS scores to include sensitive neurocognitive, functional, and quality-of-life endpoints, which are critical for informing rehabilitation strategies. Finally, incorporation of mechanistic biomarkers, such as cerebral perfusion imaging and indices of CSF dynamics, would help disentangle biological from temporal effects. Ongoing trials, including TIMELY (NCT06632587), REEL (ISRCTN14996072), and ChiCTR-TRC-12002571, are poised to address some of these questions, though their heterogenous timing definitions again underscore the urgent need for standardisation.

### Methodological considerations

This study represents the most comprehensive systematic review and meta-analysis to date comparing EC and LC following DV for TBI. Methodological rigour was ensured through protocol registration a priori, adherence to PRISMA and AMSTAR-2 standards, and formal risk-of-bias assessment using ROBINS-I and RoB 2. Pre-specified definitions of EC, LC, and ultra-EC reduced misclassification bias, while stratified subgroup analysis by implant material allowed exploration of a key effect modifier often overlooked in prior reviews. These refinements distinguish this study from previous systematic reviews, which were consistently rated “critically low” on AMSTAR-2 [2, 6, 20, 28, 47] due to lack of protocol registration, limited bias assessment, and inadequate exploration of heterogeneity. By addressing these shortcomings, our review provides the most methodologically robust synthesis to date, directly informing clinical decision-making and trial design.

Nevertheless, several limitations warrant consideration. First, follow-up was variably reported and commonly short (often ≤ 6 months), and several cohorts did not report follow-up, likely under-ascertaining late postoperative complications events (including bone-flap resorption, infection, wound dehiscence, and shunt-dependence) and pooled reoperation risk. Second, the evidence base remains limited by retrospective design, small sample sizes, and confounding by indication. Heterogeneity in this study was substantial for several endpoints, reflecting variable timing definitions, implant practices, and outcome reporting. Significant hydrocephalus findings were driven by few studies, and functional outcomes were under-reported and inconsistently measured, often restricted to crude ordinal scales, precluding meaningful synthesis of neurocognitive recovery or quality of life. Furthermore, the scarcity of randomised data and risk of confounding by indication and timing-materail collinearity (such as era- and centre-specific implant choices) limits causal inference despite stratified analyses. Finally, although the certainty of evidence for the primary EC versus LC comparisons was graded moderate, the certainty for timing-material subgroup analyses was generally low or very low due to imprecision, small study numbers, and residual confounding.

## Conclusion

This systematic review and meta-analysis, the most comprehensive to date, suggests that CP timing alone does not dictate safety or efficacy. Outcomes are instead critically modified by implant and perioperative context. Ultra-EC may offer operative and physiological advantages without a detectable increase in infection or mortality in the limited available data, but early autologous or allogenic reimplantation carries higher risks of complication and reoperation. These findings challenge the long-standing binary debate of “early” versus “late” and argue for a paradigm shift towards material- and patient-specific strategies. For clinical practice, this means considering implant choice and patient profiling alongside timing. For research, it underscores the need for harmonised definitions, material-stratified prospective trials, and inclusion of long-term functional and quality-of-life outcomes. Only through such refinement can the field move beyond controversy and establish evidence-based guidelines for optimal CP timing.

## Supplementary Information

Below is the link to the electronic supplementary material.

ESM 1 Supplementary Material 1 (DOCX 4.27 MB)

## Data Availability

All data supporting the findings of this review are contained within the article and the attached supplemental digital content. No additional dataset was generated or analysed. The review protocol is available at: https://www.crd.york.ac.uk/prospero/display_record.php?ID=CRD420251032162. Additional materials are available from the corresponding author on reasonable request.

## Abbreviations

CI: Confidence intervals
CP: Cranioplasty
CSF: Cerebrospinal fluid
DC: Decompressive Craniectomy
EC: Early Cranioplasty
GOS: Glasgow Outcome Scale
ICP: Intracranial Pressure
LC: Late Cranioplasty
MD: Mean Difference
mRS: Modified Rankin Scale
NOS: Newcastle-Ottawa Scale
RCT: Randomised-Controlled Trial
ROBINS-I: Risk of Bias in Non-Randomised Studies of—Interventions tool
RR: Risk Ratio
TBI: Traumatic Brain Injury
Ultra-EC: Ultra-Early Cranioplasty
