# Supplementary material for: Optimal timing of cranioplasty post-decompressive craniectomy in traumatic brain injury: a systematic review, meta-analysis, and overview of ongoing trials
Source: Acta Neurochir (Wien). 2026 Jan 8;168(1):7. doi: 10.1007/s00701-025-06759-2 (PMC12789150; doi:10.1007/s00701-025-06759-2)
Supplement: Supplementary file 1 — Supplementary Material 1 (DOCX 4.27 MB) [file 701_2025_6759_MOESM1_ESM.pdf]

Database	Search strategy
Pubmed/MEDLINE (n=128)	(Cranioplasty[Mesh] OR cranioplasty OR "cranial reconstruction" OR "skull reconstruction" OR "skull repair" OR "bone flap replacement")<br>AND<br>("Decompressive Craniectomy"[Mesh] OR "decompressive craniectomy" OR "decompression surgery")<br>AND<br>("Brain Injuries, Traumatic"[Mesh] OR "traumatic brain injury" OR TBI OR "head injury" OR "head trauma")<br>AND<br>(timing OR "early cranioplasty" OR "late cranioplasty" OR "delayed cranioplasty" OR early OR delayed OR late)
Embase (n=232)	(exp Cranioplasty/ OR cranioplasty OR "cranial reconstruction" OR "skull reconstruction" OR "skull repair" OR "bone flap replacement")<br>AND<br>(exp "Decompressive Craniectomy"/ OR "decompressive craniectomy" OR "decompression surgery")<br>AND<br>(exp "Brain Injuries, Traumatic"/ OR "traumatic brain injury" OR TBI OR "head injury" OR "head trauma")<br>AND<br>(timing OR "early cranioplasty" OR "late cranioplasty" OR "delayed cranioplasty" OR early OR delayed OR late)
CENTRAL (n=13)	([mh Cranioplasty] OR cranioplasty OR "cranial reconstruction" OR "skull reconstruction" OR "skull repair" OR "bone flap replacement")<br>AND<br>([mh "Decompressive Craniectomy"] OR "decompressive craniectomy" OR "decompression surgery")<br>AND<br>([mh "Brain Injuries, Traumatic"] OR "traumatic brain injury" OR TBI OR "head injury" OR "head trauma")<br>AND<br>(timing OR "early cranioplasty" OR "late cranioplasty" OR "delayed cranioplasty" OR early OR delayed OR late)

Supplementary Table 1A: Search strategies, n = number of articles returned.

Database	Query	Results	Outcome
ClinicalTrials.gov	Cranioplasty	41	NCT06632587<br>NCT03222297<br>39 NOI
International Standard Registered Clinical/soCial sTudy Number (ISRCTN)	Cranioplasty	8	ISRCTN14996072<br>7 NOI
WHO International Clinical Trials Registry Platform (ICTRP)	Cranioplasty	69	ChiCTR-TRC-12002571<br>68 NOI
Australian New Zealand Clinical Trials Registry (ANZCTR)	Cranioplasty	5	5 NOI
The Brazilian Registry of Clinical Trials (ReBEC)	Cranioplasty	2	2 NOI
Chinese Clinical Trial Registry (ChiCTR)	Cranioplasty	13	2 duplicates<br>11 NOI
Korean the Clinical Research Information Service (CRIS)	Cranioplasty	0	-
The Clinical Trials Registry - India (CTRI)	Cranioplasty	0	-
Cuban Public Registry of Clinical Trials (RPCEC)	Cranioplasty	0	-
EU Clinical Trials Register (EU-CTR)	Cranioplasty	6	6 NOI
German Clinical Trials Register (DRKS)	Cranioplasty	7	7 NOI
International Traditional Medicine Clinical Trial Registry (ITMCTR)	Cranioplasty	0	-
Japan Registry for Clinical Trials (jRCT)	Cranioplasty	2	2 NOI
Lebanese Clinical Trail Registry (LBCTR)	Cranioplasty	0	-
Thai Clinical Trials Registry (TCTR)	Cranioplasty	2	2 NOI
Pan African Clinical Trials Registry (PACTR)	Cranioplasty	0	-
Sri Lanka Trials Registry (SLCTR)	Cranioplasty	0	-

Supplementary Table 1B: Search strategies used for unpublished RCTs.

Abbreviations: NOI = No outcomes of interest; RCT = randomised control trial.

No.	Item	Outcome
1	Did the research questions and inclusion criteria for the review include the components of PICO?	Y
2	*Did the report of the review contain an explicit statement that the review methods were established prior to the conduct of the review and did the report justify any significant deviations from the protocol?	Y
3	Did the review authors explain their selection of the study designs for inclusion in the review?	Y
4	*Did the review authors use a comprehensive literature search strategy?	Y
5	Did the review authors perform study selection in duplicate?	Y
6	Did the review authors perform data extraction in duplicate?	Y
7	*Did the review authors provide a list of excluded studies and justify the exclusions?	Y
8	Did the review authors describe the included studies in adequate detail?	Y
9	*Did the review authors use a satisfactory technique for assessing the risk of bias (RoB) in individual studies that were included in the review?	Y
10	Did the review authors report on the sources of funding for the studies included in the review?	Y
11	*If meta-analysis was performed did the review authors use appropriate methods for statistical combination of results?	Y
12	If meta-analysis was performed, did the review authors assess the potential impact of RoB in individual studies on the results of the meta-analysis or other evidence synthesis?	Y
13	*Did the review authors account for RoB in individual studies when interpreting/ discussing the results of the review?	Y
14	Did the review authors provide a satisfactory explanation for, and discussion of, any heterogeneity observed in the results of the review?	Y
15	*If they performed quantitative synthesis did the review authors carry out an adequate investigation of publication bias (small study bias) and discuss its likely impact on the results of the review?	Y
16	Did the review authors report any potential sources of conflict of interest, including any funding they received for conducting the review?	Y

Supplementary Table 2: AMSTAR 2 criteria for our present review. *= *Critical weaknesses*

Study or Subgroup	EC Events	EC Total	LC Events	LC Total	Weight	Risk ratio IV, Random, 95% CI
**1.3.1 Mixed bone flap material use**						
Cho (2011)	2	15	1	21	8.0%	2.80 [0.28 , 28.13]
Eaton (2022)	5	141	7	294	12.0%	1.49 [0.48 , 4.61]
Piedra (2014)	6	78	1	79	8.7%	6.08 [0.75 , 49.32]
Tora (2021)	8	51	2	91	10.7%	7.14 [1.57 , 32.34]
Vreeburg (2024)	9	73	4	100	12.0%	3.08 [0.99 , 9.62]
Vyas and Singh (2021)	0	42	3	46	6.3%	0.16 [0.01 , 2.94]
**Subtotal (Wald[a])**		**400**		**631**	**57.7%**	**2.63 [1.35 , 5.12]**
Total events:	30		18			
Test for overall effect: Z = 2.84 (P = 0.005)						
Heterogeneity: Tau² (REML[b]) = 0.06; Chi² = 6.90, df = 5 (P = 0.23); I² = 8%						
**1.3.2 Only synthetic bone flap material use**						
Songara (2016)	0	6	2	10	6.4%	0.31 [0.02 , 5.63]
**Subtotal**		**6**		**10**	**6.4%**	**0.31 [0.02 , 5.63]**
Total events:	0		2			
Test for overall effect: Z = 0.79 (P = 0.43)						
Heterogeneity: Not applicable						
**1.3.3 Only autologous or allogenic bone flap material use**						
Nasi (2018)	3	59	34	71	12.0%	0.11 [0.03 , 0.33]
Sethi (2022)	4	39	12	38	12.3%	0.32 [0.11 , 0.92]
Yang (2018)	2	15	64	144	11.4%	0.30 [0.08 , 1.10]
**Subtotal (Wald[a])**		**113**		**253**	**35.8%**	**0.22 [0.10 , 0.45]**
Total events:	9		110			
Test for overall effect: Z = 4.09 (P < 0.0001)						
Heterogeneity: Tau² (REML[b]) = 0.08; Chi² = 2.36, df = 2 (P = 0.31); I² = 18%						
**Total (Wald[a])**		**519**		**894**	**100.0%**	**0.90 [0.34 , 2.41]**
Total events:	39		130			
Test for overall effect: Z = 0.21 (P = 0.83)						
Test for subgroup differences: Chi² = 24.79, df = 2 (P < 0.00001), I² = 91.9%						
Heterogeneity: Tau² (REML[b]) = 1.76; Chi² = 38.29, df = 9 (P < 0.0001); I² = 76%						

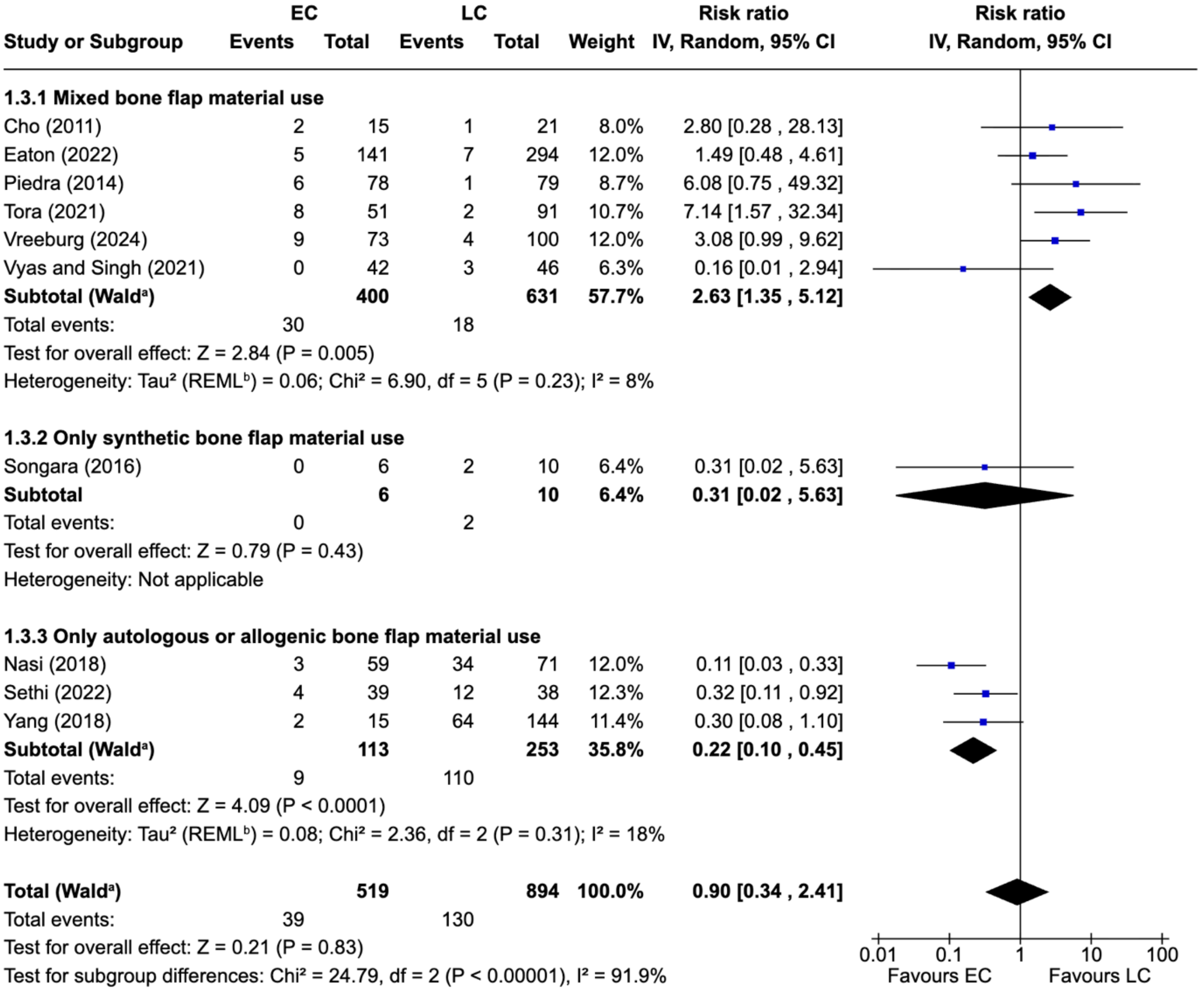

Footnotes
[a]CI calculated by Wald-type method.
[b]Tau² calculated by Restricted Maximum-Likelihood method.

Supplementary Figure 1A: Hydrocephalus with bone flap material type subgroup analysis.

Study or Subgroup	Ultra-EC Events	Ultra-EC Total	Other Events	Other Total	Weight	Risk ratio IV, Random, 95% CI
Sethi (2022)	4	39	12	38	61.1%	0.32 [0.11 , 0.92]
Yang (2018)	2	15	64	144	38.9%	0.30 [0.08 , 1.10]
**Total (Wald[a])**		**54**		**182**	**100.0%**	**0.31 [0.14 , 0.71]**
Total events:	6		76			
Test for overall effect: Z = 2.79 (P = 0.005)						
Heterogeneity: Tau² (REML[b]) = 0.00; Chi² = 0.01, df = 1 (P = 0.93); I² = 0%						

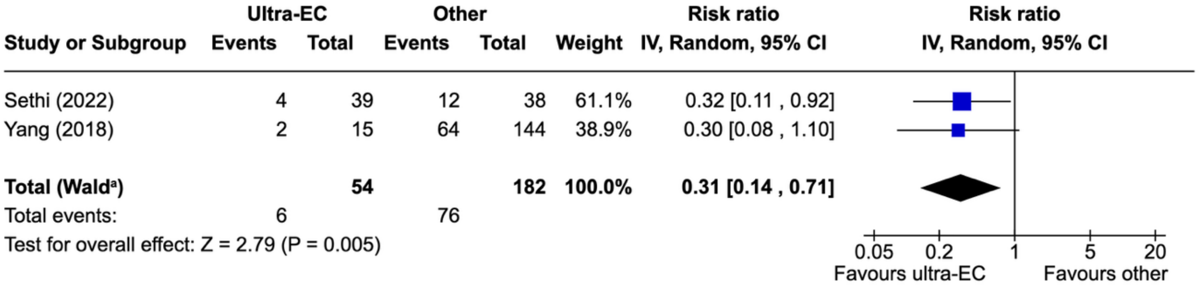

Footnotes
[a]CI calculated by Wald-type method.
[b]Tau² calculated by Restricted Maximum-Likelihood method.

Supplementary Figure 1B: Hydrocephalus with ultra-EC subgroup analysis.

Study or Subgroup	EC Events	EC Total	LC Events	LC Total	Weight	Risk ratio IV, Random, 95% CI
Nasi (2018)	0	59	34	71	22.4%	0.02 [0.00 , 0.28]
Piedra (2014)	6	78	1	79	25.0%	6.08 [0.75 , 49.32]
Tora (2021)	8	51	2	91	27.0%	7.14 [1.57 , 32.34]
Yang (2018)	1	15	29	144	25.6%	0.33 [0.05 , 2.26]
**Total (Wald[a])**		**203**		**385**	**100.0%**	**0.81 [0.06 , 11.73]**
Total events:	15		66			

Test for overall effect: $Z = 0.15$ ($P = 0.88$)

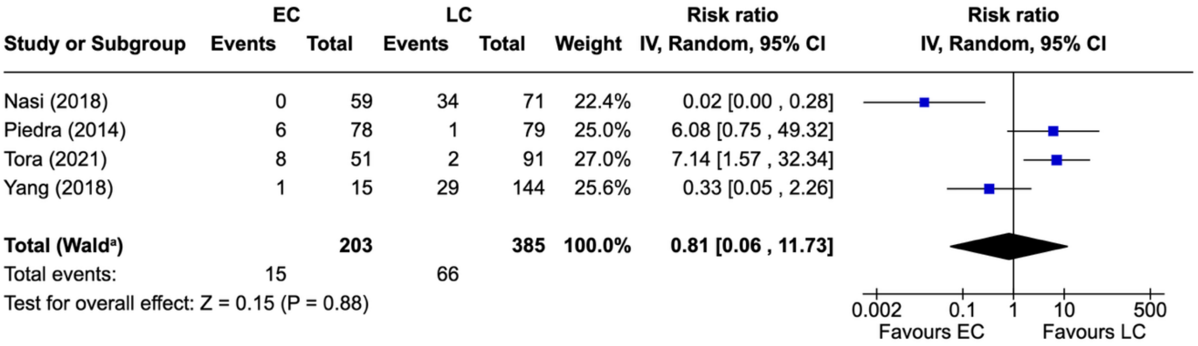

Heterogeneity: Tau^2 (REML[b]) = 6.29; $\text{Chi}^2 = 18.13$, df = 3 ($P = 0.0004$); $I^2 = 86\%$

Footnotes

[a]CI calculated by Wald-type method.

[b]Tau^2 calculated by Restricted Maximum-Likelihood method.

Supplementary Figure 1C: Hydrocephalus requiring shunting.

Study or Subgroup	EC Events	EC Total	LC Events	LC Total	Weight	Risk ratio IV, Random, 95% CI
**1.5.1 Mixed bone flap material use**						
Chaturhvedi (2015)	0	20	0	54		Not estimable
Cho (2011)	1	15	2	21	5.6%	0.70 [0.07 , 7.03]
Jiang and Wang (2020)	5	28	5	32	13.7%	1.14 [0.37 , 3.54]
Tora (2021)	6	51	1	91	6.5%	10.71 [1.33 , 86.47]
**Subtotal (Wald[a])**		**114**		**198**	**25.7%**	**1.89 [0.44 , 8.14]**
Total events:	12		8			
Test for overall effect: Z = 0.86 (P = 0.39)						
Heterogeneity: Tau² (REML[b]) = 0.84; Chi² = 4.01, df = 2 (P = 0.13); I² = 50%						
**1.5.2 Only synthetic bone flap material use**						
Chun and Yi (2011)	2	30	7	15	10.6%	0.14 [0.03 , 0.61]
Song (2014)	2	25	3	18	8.7%	0.48 [0.09 , 2.58]
Zhang (2010)	1	23	2	47	5.4%	1.02 [0.10 , 10.69]
**Subtotal (Wald[a])**		**78**		**80**	**24.7%**	**0.32 [0.11 , 0.99]**
Total events:	5		12			
Test for overall effect: Z = 1.97 (P = 0.05)						
Heterogeneity: Tau² (REML[b]) = 0.18; Chi² = 2.36, df = 2 (P = 0.31); I² = 18%						
**1.5.3 Only autologous or allogenic bone flap material use**						
Nasi (2018)	8	59	25	71	18.9%	0.39 [0.19 , 0.79]
Schuss (2012)	1	54	2	226	5.3%	2.09 [0.19 , 22.66]
Sethi (2022)	0	39	2	38	3.6%	0.20 [0.01 , 3.93]
Yang (2018)	8	15	91	144	21.9%	0.84 [0.52 , 1.38]
**Subtotal (Wald[a])**		**167**		**479**	**49.6%**	**0.62 [0.32 , 1.20]**
Total events:	17		120			
Test for overall effect: Z = 1.41 (P = 0.16)						
Heterogeneity: Tau² (REML[b]) = 0.16; Chi² = 4.67, df = 3 (P = 0.20); I² = 39%						
**Total (Wald[a])**		**359**		**757**	**100.0%**	**0.70 [0.38 , 1.30]**
Total events:	34		140			
Test for overall effect: Z = 1.13 (P = 0.26)						
Test for subgroup differences: Chi² = 3.53, df = 2 (P = 0.17), I² = 43.4%						
Heterogeneity: Tau² (REML[b]) = 0.39; Chi² = 16.94, df = 9 (P = 0.05); I² = 50%						

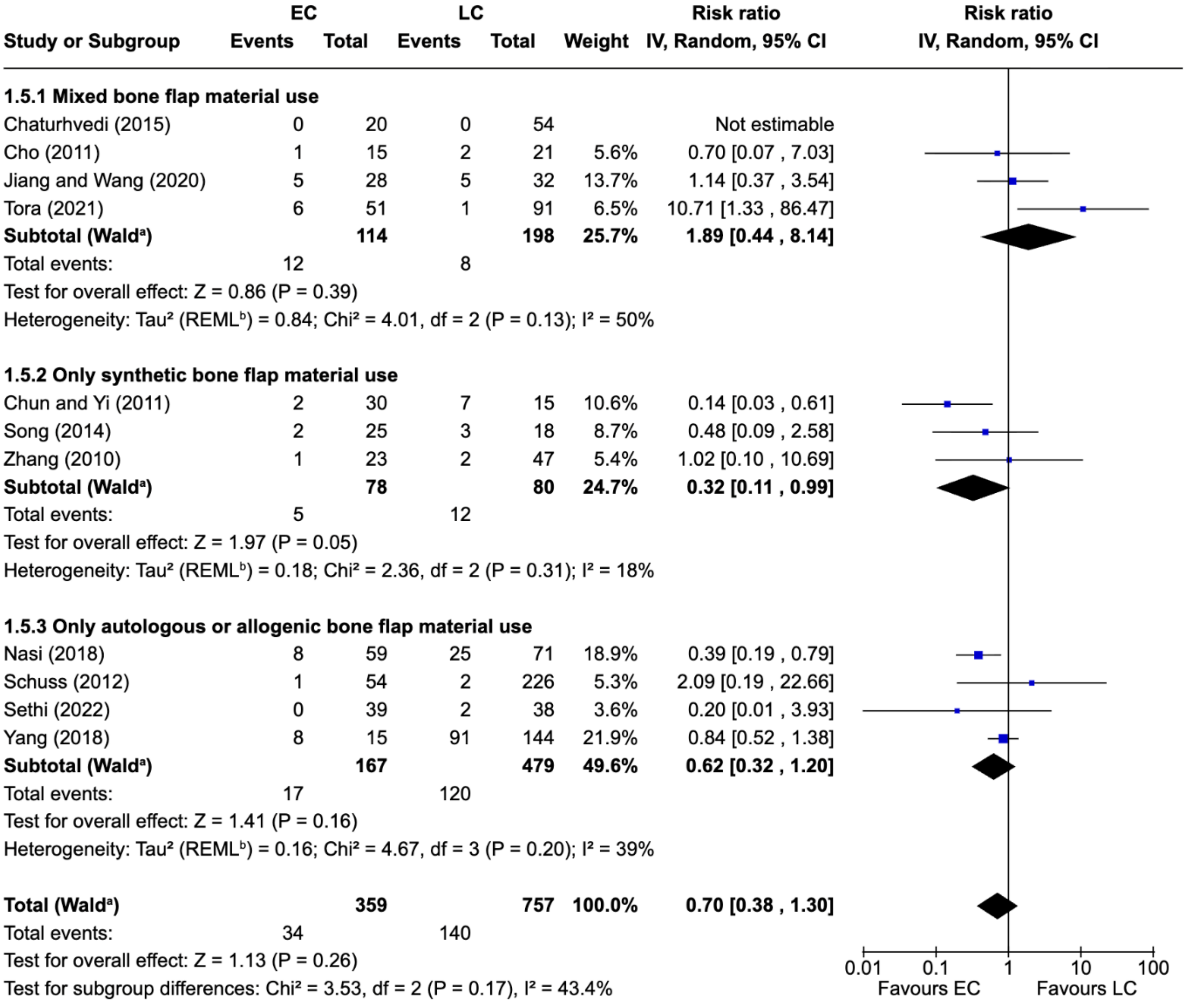

Footnotes
[a]CI calculated by Wald-type method.
[b]Tau² calculated by Restricted Maximum-Likelihood method.

Supplementary Figure 2A: Extra-axial fluid collection with bone flap material type subgroup analysis.

Study or Subgroup	Ultra-EC Events	Ultra-EC Total	Other Events	Other Total	Weight	Risk ratio IV, Random, 95% CI
Chun and Yi (2011)	2	30	7	15	33.9%	0.14 [0.03 , 0.61]
Sethi (2022)	0	39	2	38	15.0%	0.20 [0.01 , 3.93]
Yang (2018)	8	15	91	144	51.1%	0.84 [0.52 , 1.38]
**Total (Wald[a])**		**84**		**197**	**100.0%**	**0.37 [0.10 , 1.45]**
Total events:	10		100			
Test for overall effect: Z = 1.43 (P = 0.15)						
Heterogeneity: Tau² (REML[b]) = 0.88; Chi² = 5.90, df = 2 (P = 0.05); I² = 64%						

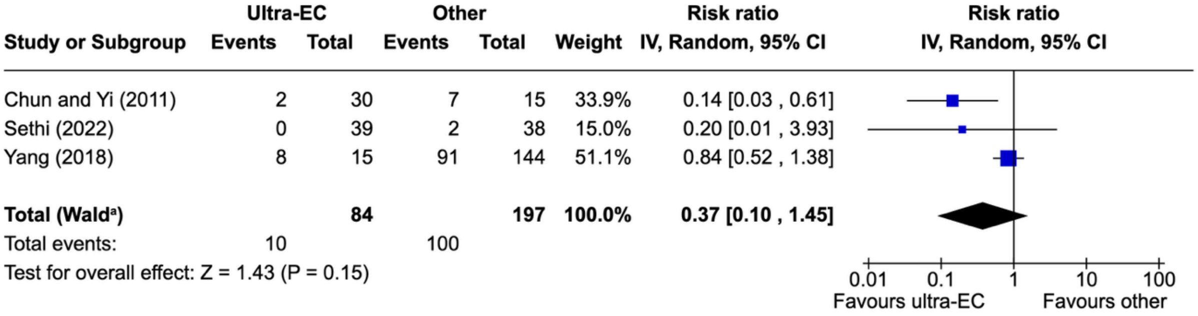

Footnotes
[a]CI calculated by Wald-type method.
[b]Tau² calculated by Restricted Maximum-Likelihood method.

Supplementary Figure 2B: Extra-axial fluid collection with ultra-EC subgroup analysis.

Study or Subgroup	EC Events	EC Total	LC Events	LC Total	Weight	Risk ratio IV, Random, 95% CI
**1.1.1 Mixed bone flap material use**						
Chaturhvedi (2015)	1	20	7	54	4.0%	0.39 [0.05 , 2.94]
Cho (2011)	0	15	0	21		Not estimable
Eaton (2022)	11	141	15	294	22.4%	1.53 [0.72 , 3.24]
Jiang and Wang (2020)	6	28	6	32	14.0%	1.14 [0.42 , 3.14]
Piedra (2014)	6	78	11	79	15.7%	0.55 [0.21 , 1.42]
Tora (2021)	5	51	2	91	6.2%	4.46 [0.90 , 22.17]
Vyas and Singh (2021)	2	44	0	46	1.9%	5.22 [0.26 , 105.81]
**Subtotal (Wald[a])**		**377**		**617**	**64.3%**	**1.19 [0.65 , 2.18]**
Total events:	31		41			
Test for overall effect: Z = 0.58 (P = 0.57)						
Heterogeneity: Tau² (REML[b]) = 0.16; Chi² = 7.69, df = 5 (P = 0.17); I² = 30%						
**1.1.2 Only synthetic bone flap material use**						
Chun and Yi (2011)	0	30	1	15	1.7%	0.17 [0.01 , 3.99]
Song (2014)	0	25	0	18		Not estimable
Songara (2016)	0	6	0	10		Not estimable
Zhang (2010)	0	23	0	47		Not estimable
Zhu (2018)	1	97	0	100	1.7%	3.09 [0.13 , 74.98]
**Subtotal (Wald[a])**		**181**		**190**	**3.4%**	**0.72 [0.04 , 12.21]**
Total events:	1		1			
Test for overall effect: Z = 0.23 (P = 0.82)						
Heterogeneity: Tau² (REML[b]) = 1.56; Chi² = 1.60, df = 1 (P = 0.21); I² = 37%						
**1.1.3 Only autologous or allogenic bone flap material use**						
Kumar (2018)	0	21	0	21		Not estimable
Nasi (2018)	4	59	9	71	11.7%	0.53 [0.17 , 1.65]
Schuss (2012)	1	54	3	226	3.3%	1.40 [0.15 , 13.15]
Sethi (2022)	5	39	7	38	13.0%	0.70 [0.24 , 2.00]
Yang (2018)	1	15	17	144	4.3%	0.56 [0.08 , 3.95]
**Subtotal (Wald[a])**		**188**		**500**	**32.4%**	**0.66 [0.33 , 1.30]**
Total events:	11		36			
Test for overall effect: Z = 1.21 (P = 0.23)						
Heterogeneity: Tau² (REML[b]) = 0.00; Chi² = 0.60, df = 3 (P = 0.90); I² = 0%						
**Total (Wald[a])**		**746**		**1307**	**100.0%**	**0.96 [0.63 , 1.46]**
Total events:	43		78			
Test for overall effect: Z = 0.19 (P = 0.85)						
Test for subgroup differences: Chi² = 1.68, df = 2 (P = 0.43), I² = 0%						
Heterogeneity: Tau² (REML[b]) = 0.05; Chi² = 11.87, df = 11 (P = 0.37); I² = 10%						

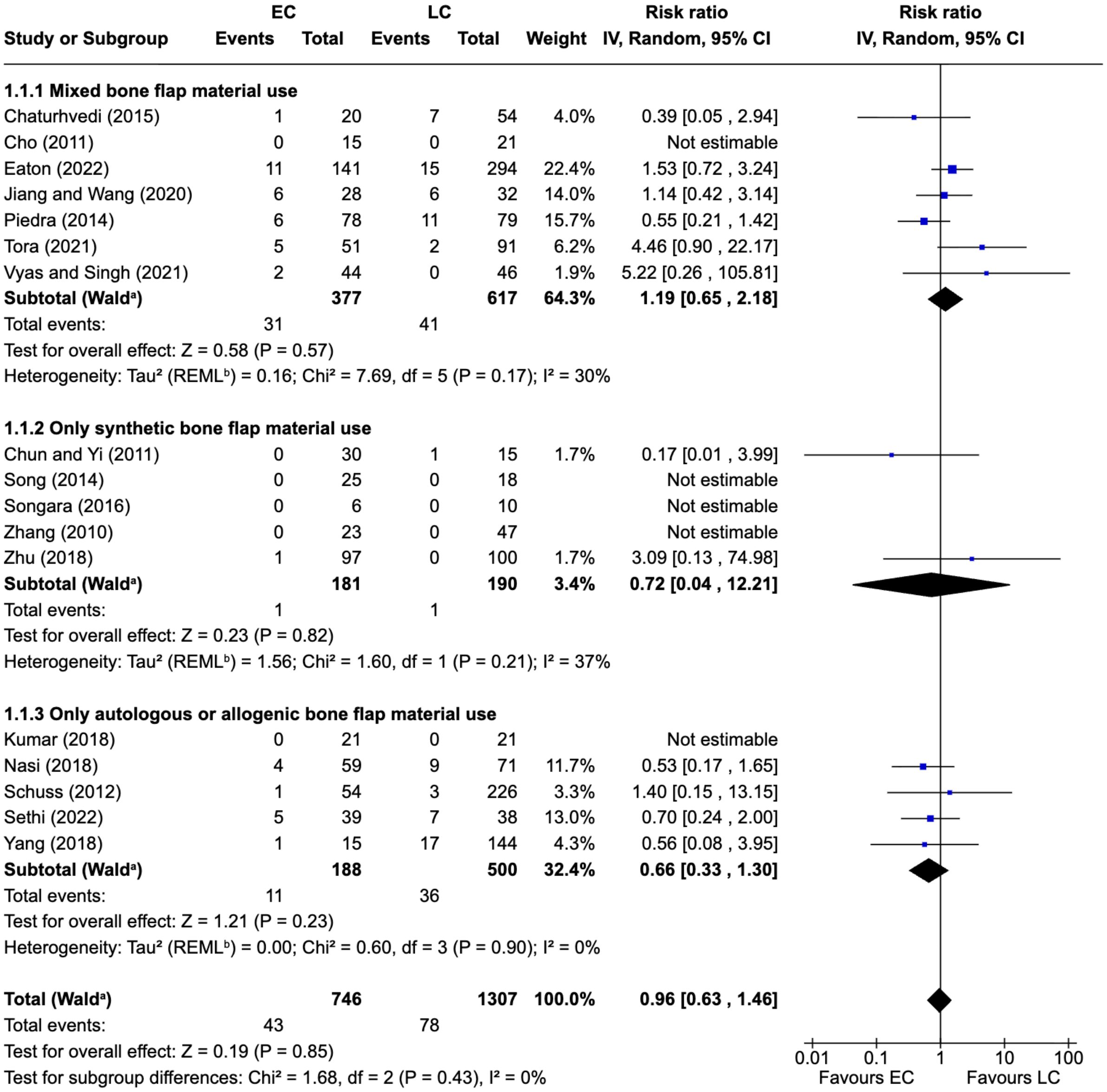

Footnotes

[a]CI calculated by Wald-type method.

[b]Tau² calculated by Restricted Maximum-Likelihood method.

Supplementary Figure 3A: Infection with bone flap material type subgroup analysis.

Study or Subgroup	Ultra-EC Events	Ultra-EC Total	Other Events	Other Total	Weight	Risk ratio IV, Random, 95% CI
Chun and Yi (2011)	0	30	1	15	8.0%	0.17 [0.01 , 3.99]
Sethi (2022)	5	39	7	38	71.0%	0.70 [0.24 , 2.00]
Yang (2018)	1	15	17	144	21.0%	0.56 [0.08 , 3.95]
**Total (Wald[a])**		**84**		**197**	**100.0%**	**0.60 [0.24 , 1.45]**
Total events:	6		25			

Test for overall effect: Z = 1.14 (P = 0.25)

Heterogeneity: Tau² (REML[b]) = 0.00; Chi² = 0.69, df = 2 (P = 0.71); I² = 0%

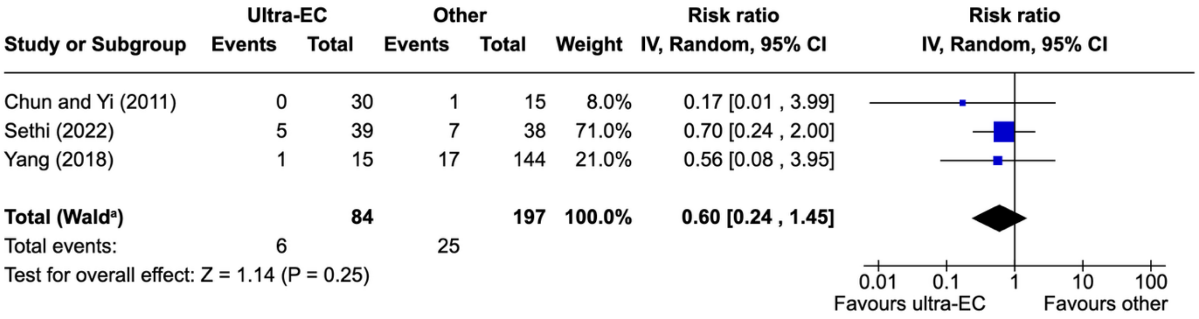

Footnotes

[a]CI calculated by Wald-type method.

[b]Tau² calculated by Restricted Maximum-Likelihood method.

Supplementary Figure 3B: Infection with ultra-EC subgroup analysis.

Study or Subgroup	EC Events	EC Total	LC Events	LC Total	Weight	Risk ratio IV, Random, 95% CI
**1.2.1 Mixed bone flap material use**						
Eaton (2022)	5	141	10	294	23.5%	1.04 [0.36 , 2.99]
Jiang and Wang (2020)	5	28	7	32	24.7%	0.82 [0.29 , 2.29]
Piedra (2014)	3	78	1	79	5.2%	3.04 [0.32 , 28.58]
Vyas and Singh (2021)	0	42	1	46	2.6%	0.36 [0.02 , 8.71]
**Subtotal (Wald[a])**		**289**		**451**	**56.0%**	**0.98 [0.50 , 1.95]**
Total events:	13		19			
Test for overall effect: Z = 0.04 (P = 0.96)						
Heterogeneity: Tau² (REML[b]) = 0.00; Chi² = 1.49, df = 3 (P = 0.69); I² = 0%						
**1.2.2 Only synthetic bone flap material use**						
Chun and Yi (2011)	0	30	0	15		Not estimable
Songara (2016)	0	6	0	10		Not estimable
**Subtotal**		**36**		**25**		**Not estimable**
Total events:	0		0			
Test for overall effect: Not applicable						
Heterogeneity: Not applicable						
**1.2.3 Only autologous or allogenic bone flap material use**						
Kumar (2018)	2	21	0	21	3.0%	5.00 [0.25 , 98.27]
Schuss (2012)	3	54	14	226	17.8%	0.90 [0.27 , 3.01]
Yang (2018)	3	15	29	144	23.2%	0.99 [0.34 , 2.88]
**Subtotal (Wald[a])**		**90**		**391**	**44.0%**	**1.06 [0.49 , 2.30]**
Total events:	8		43			
Test for overall effect: Z = 0.15 (P = 0.88)						
Heterogeneity: Tau² (REML[b]) = 0.00; Chi² = 1.13, df = 2 (P = 0.57); I² = 0%						
**Total (Wald[a])**		**415**		**867**	**100.0%**	**1.02 [0.61 , 1.70]**
Total events:	21		62			

Test for overall effect: Z = 0.07 (P = 0.95)

Test for subgroup differences: Chi² = 0.02, df = 1 (P = 0.89), I² = 0%

Heterogeneity: Tau² (REML[b]) = 0.00; Chi² = 2.64, df = 6 (P = 0.85); I² = 0%

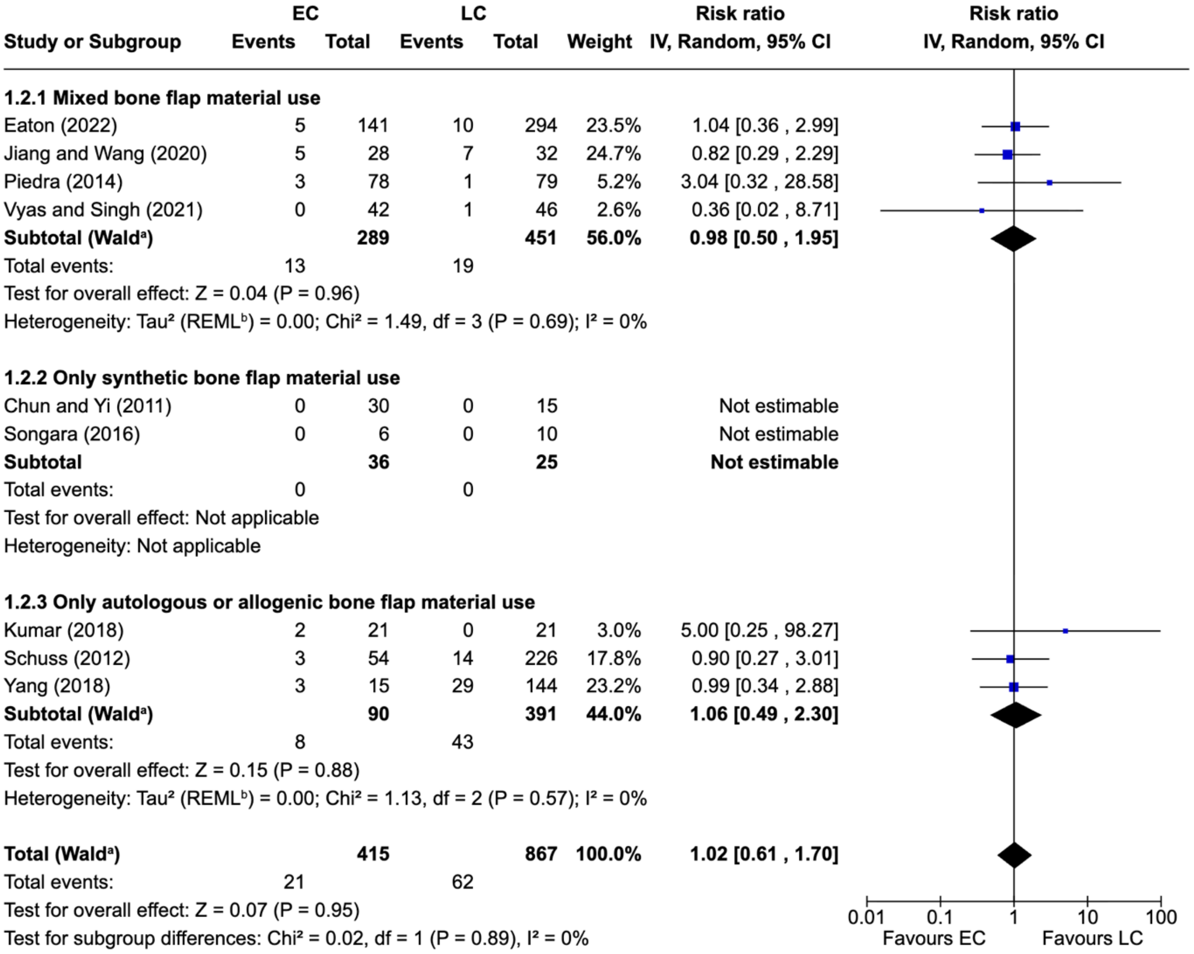

Footnotes

[a]CI calculated by Wald-type method.

[b]Tau² calculated by Restricted Maximum-Likelihood method.

Supplementary Figure 4A: Haematoma with bone flap material type subgroup analysis.

Study or Subgroup	Ultra-EC Events	Ultra-EC Total	Other Events	Other Total	Weight	Risk ratio IV, Random, 95% CI
Chun and Yi (2011)	0	30	0	15		Not estimable
Yang (2018)	3	15	29	144	100.0%	0.99 [0.34 , 2.88]
**Total**		**45**		**159**	**100.0%**	**0.99 [0.34 , 2.88]**
Total events:	3		29			

Test for overall effect: Z = 0.01 (P = 0.99)

Heterogeneity: Not applicable

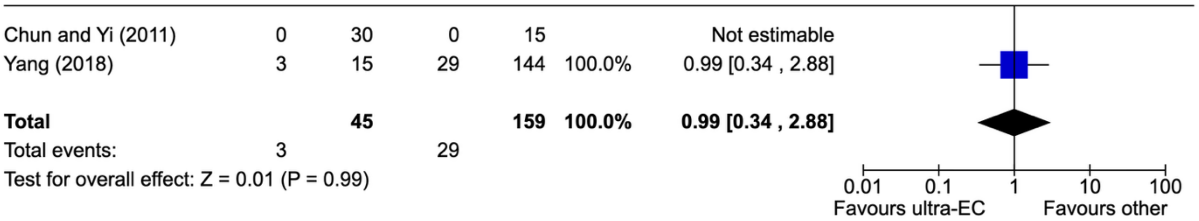

Supplementary Figure 4B: Haematoma with ultra-EC subgroup analysis.

Study or Subgroup	EC Events	EC Total	LC Events	LC Total	Weight	Risk ratio IV, Random, 95% CI
Piedra (2014)	12	78	15	79	83.5%	0.81 [0.41 , 1.62]
Sethi (2022)	0	39	1	38	4.0%	0.33 [0.01 , 7.74]
Tora (2021)	1	51	4	91	8.5%	0.45 [0.05 , 3.88]
Vyas and Singh (2021)	0	42	1	46	4.0%	0.36 [0.02 , 8.71]
**Total (Wald[a])**		**210**		**254**	**100.0%**	**0.72 [0.38 , 1.35]**
Total events:	13		21			

Test for overall effect: Z = 1.02 (P = 0.31)

Heterogeneity: Tau² (REML[b]) = 0.00; Chi² = 0.72, df = 3 (P = 0.87); I² = 0%

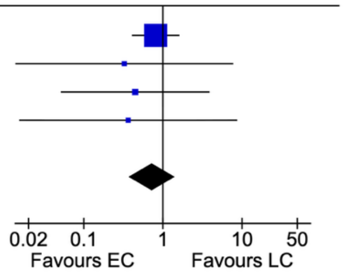

Footnotes

[a]CI calculated by Wald-type method.

[b]Tau² calculated by Restricted Maximum-Likelihood method.

Supplementary Figure 5: Bone resorption.

Study or Subgroup	EC Events	EC Total	LC Events	LC Total	Weight	Risk ratio IV, Random, 95% CI
**1.7.1 Mixed bone flap material use**						
Chaturhvedi (2015)	2	20	3	54	11.0%	1.80 [0.32 , 9.99]
Eaton (2022)	5	141	15	294	31.3%	0.70 [0.26 , 1.87]
Vreeburg (2024)	11	73	9	100	43.8%	1.67 [0.73 , 3.83]
**Subtotal (Wald[a])**		**234**		**448**	**86.1%**	**1.22 [0.63 , 2.38]**
Total events:	18		27			
Test for overall effect: Z = 0.59 (P = 0.55)						
Heterogeneity: Tau^2 (REML[b]) = 0.06; Chi^2 = 1.99, df = 2 (P = 0.37); I^2 = 16%						
**1.7.2 Only synthetic bone flap material use**						
Songara (2016)	0	6	2	10	3.9%	0.31 [0.02 , 5.63]
Zhang (2010)	1	23	3	47	6.7%	0.68 [0.07 , 6.19]
**Subtotal (Wald[a])**		**29**		**57**	**10.6%**	**0.51 [0.09 , 2.95]**
Total events:	1		5			
Test for overall effect: Z = 0.75 (P = 0.45)						
Heterogeneity: Tau^2 (REML[b]) = 0.00; Chi^2 = 0.17, df = 1 (P = 0.68); I^2 = 0%						
**1.7.3 Only autologous or allogenic bone flap material use**						
Kumar (2018)	1	21	0	21	3.3%	3.00 [0.13 , 69.70]
**Subtotal**		**21**		**21**	**3.3%**	**3.00 [0.13 , 69.70]**
Total events:	1		0			
Test for overall effect: Z = 0.68 (P = 0.49)						
Heterogeneity: Not applicable						
**Total (Wald[a])**		**284**		**526**	**100.0%**	**1.15 [0.65 , 2.04]**
Total events:	20		32			
Test for overall effect: Z = 0.48 (P = 0.63)						
Test for subgroup differences: Chi^2 = 1.21, df = 2 (P = 0.55), I^2 = 0%						
Heterogeneity: Tau^2 (REML[b]) = 0.02; Chi^2 = 3.39, df = 5 (P = 0.64); I^2 = 3%						

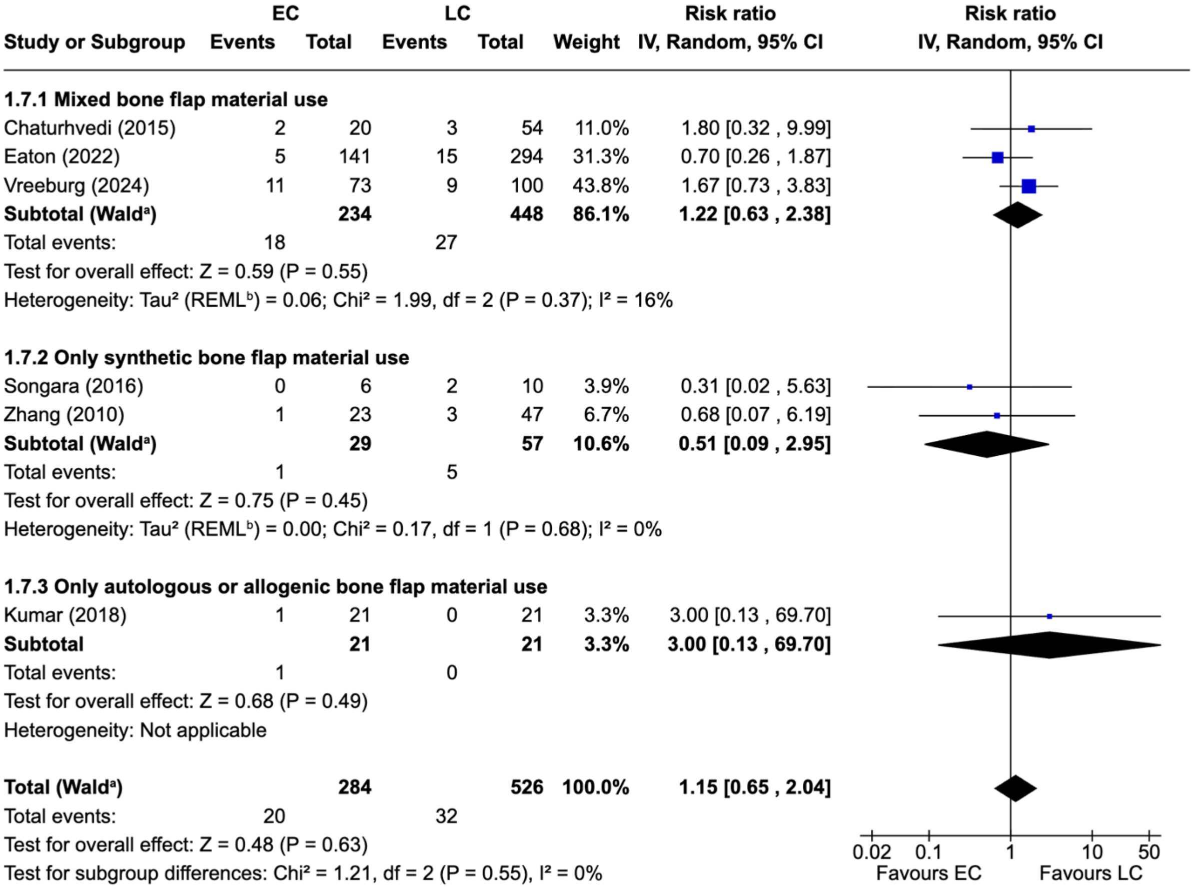

Footnotes

[a]CI calculated by Wald-type method.

[b]Tau^2 calculated by Restricted Maximum-Likelihood method.

Supplementary Figure 6: Seizures with bone flap material type subgroup analysis.

Study or Subgroup	EC Events	EC Total	LC Events	LC Total	Weight	Risk ratio IV, Random, 95% CI
**1.9.1 Mixed bone flap material use**						
Chaturhvedi (2015)	1	20	0	54	4.0%	7.86 [0.33 , 185.36]
Cho (2011)	0	15	0	21		Not estimable
Eaton (2022)	0	141	0	294		Not estimable
Piedra (2014)	0	78	0	79		Not estimable
Tora (2021)	0	51	0	91		Not estimable
Vreeburg (2024)	4	73	3	100	18.8%	1.83 [0.42 , 7.91]
Vyas and Singh (2021)	0	44	0	46		Not estimable
**Subtotal (Wald[a])**		**422**		**685**	**22.9%**	**2.36 [0.63 , 8.94]**
Total events:	5		3			
Test for overall effect: Z = 1.27 (P = 0.20)						
Heterogeneity: Tau² (REML[b]) = 0.00; Chi² = 0.67, df = 1 (P = 0.41); I² = 0%						
**1.9.2 Only synthetic bone flap material use**						
Chun and Yi (2011)	0	30	0	15		Not estimable
Song (2014)	0	25	0	18		Not estimable
Songara (2016)	0	6	0	10		Not estimable
Zhu (2018)	7	97	7	100	39.7%	1.03 [0.38 , 2.83]
**Subtotal**		**158**		**143**	**39.7%**	**1.03 [0.38 , 2.83]**
Total events:	7		7			
Test for overall effect: Z = 0.06 (P = 0.95)						
Heterogeneity: Not applicable						
**1.9.3 Only autologous or allogenic bone flap material use**						
Kumar (2018)	1	21	0	21	4.1%	3.00 [0.13 , 69.70]
Schuss (2012)	0	54	0	226		Not estimable
Yang (2018)	3	15	18	144	33.4%	1.60 [0.53 , 4.81]
**Subtotal (Wald[a])**		**90**		**391**	**37.5%**	**1.71 [0.61 , 4.84]**
Total events:	4		18			
Test for overall effect: Z = 1.02 (P = 0.31)						
Heterogeneity: Tau² (REML[b]) = 0.00; Chi² = 0.14, df = 1 (P = 0.71); I² = 0%						
**Total (Wald[a])**		**670**		**1219**	**100.0%**	**1.51 [0.80 , 2.85]**
Total events:	16		28			
Test for overall effect: Z = 1.27 (P = 0.21)						
Test for subgroup differences: Chi² = 1.04, df = 2 (P = 0.59), I² = 0%						
Heterogeneity: Tau² (REML[b]) = 0.00; Chi² = 1.85, df = 4 (P = 0.76); I² = 0%						

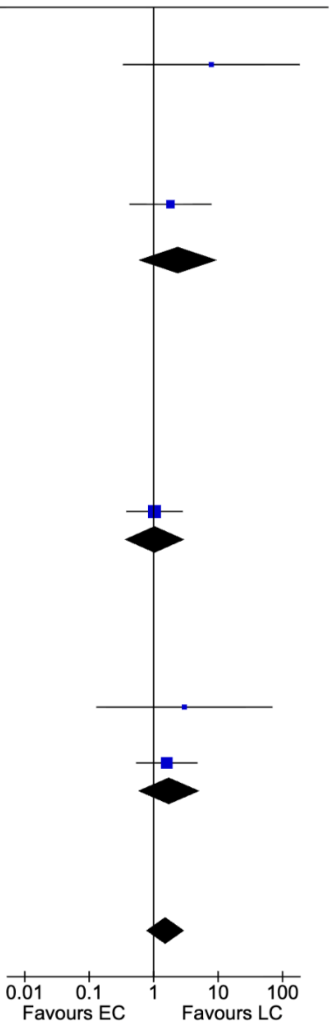

Footnotes
[a]CI calculated by Wald-type method.
[b]Tau² calculated by Restricted Maximum-Likelihood method.

Supplementary Figure 7A: Mortality with bone flap material type subgroup analysis.

Study or Subgroup	Ultra-EC Events	Ultra-EC Total	Other Events	Other Total	Weight	Risk ratio IV, Random, 95% CI
Chun and Yi (2011)	0	30	0	15		Not estimable
Sethi (2022)	3	15	18	144	100.0%	1.60 [0.53 , 4.81]
**Total**		**45**		**159**	**100.0%**	**1.60 [0.53 , 4.81]**
Total events:	3		18			
Test for overall effect: Z = 0.84 (P = 0.40)						
Heterogeneity: Not applicable						

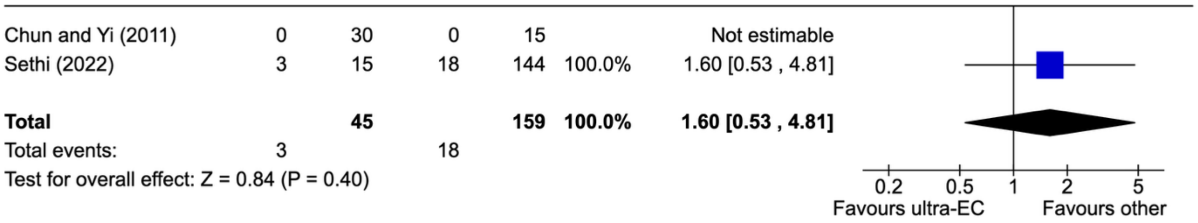

Supplementary Figure 7B: Mortality with ultra-EC subgroup analysis.

Study or Subgroup	EC Mean	EC SD	EC Total	LC Mean	LC SD	LC Total	Weight	Mean difference IV, Random, 95% CI
Chun and Yi (2011)	96.4	6.3	30	139.3	17.5	15	23.6%	-42.90 [-52.04 , -33.76]
Piedra (2014)	102	45	78	125	71	79	18.0%	-23.00 [-41.57 , -4.43]
Schuss (2012)	110	40	54	116	35	226	22.2%	-6.00 [-17.60 , 5.60]
Sethi (2022)	144	42.6	39	180	97.8	38	10.4%	-36.00 [-69.85 , -2.15]
Vyas and Singh (2021)	59.39	7.3	44	77.28	9.9	46	25.8%	-17.89 [-21.47 , -14.31]
**Total (Wald[a])**			**245**			**404**	**100.0%**	**-23.94 [-37.94 , -9.95]**

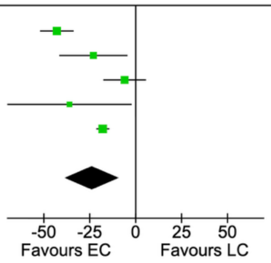

Test for overall effect: Z = 3.35 (P = 0.0008)

Heterogeneity: Tau² (REML[b]) = 194.05; Chi² = 31.97, df = 4 (P < 0.00001); I² = 88%

Footnotes
[a]CI calculated by Wald-type method.
[b]Tau² calculated by Restricted Maximum-Likelihood method.

Supplementary Figure 8A: Operative time

Study or Subgroup	Ultra-EC Mean	Ultra-EC SD	Ultra-EC Total	Other Mean	Other SD	Other Total	Weight	Mean difference IV, Random, 95% CI
Chun and Yi (2011)	96.4	6.3	30	139.3	17.5	15	93.2%	-42.90 [-52.04 , -33.76]
Sethi (2022)	144	42.6	39	180	97.8	38	6.8%	-36.00 [-69.85 , -2.15]
**Total (Wald[a])**			**69**			**53**	**100.0%**	**-42.43 [-51.25 , -33.61]**

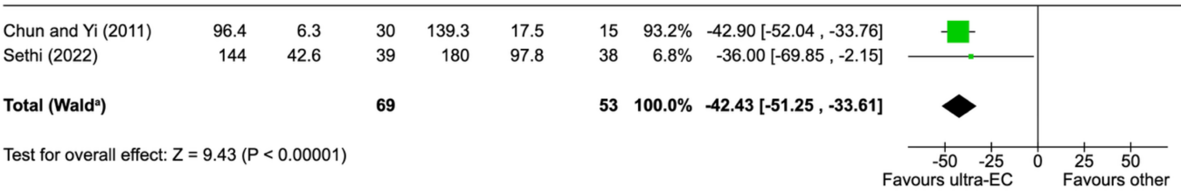

Test for overall effect: Z = 9.43 (P < 0.00001)

Heterogeneity: Tau² (REML[b]) = 0.00; Chi² = 0.15, df = 1 (P = 0.70); I² = 0%

Footnotes
[a]CI calculated by Wald-type method.
[b]Tau² calculated by Restricted Maximum-Likelihood method.

Supplementary Figure 8B: Operative time with ultra-EC subgroup analysis.

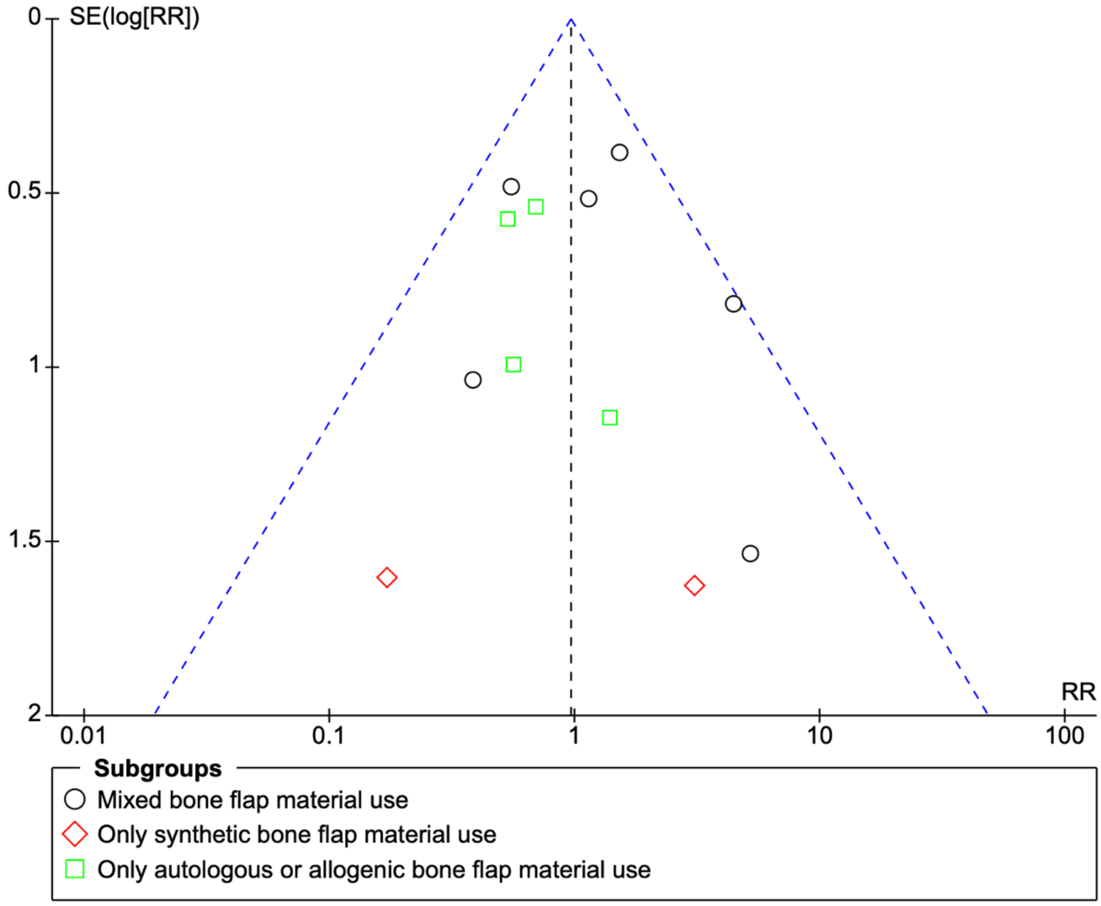

Supplementary Figure 9: Funnel plot investigating for publication bias.

Study	D1	D2	D3	D4	D5	D6	D7	Overall
Chaturhvedi et al., 2015	X	-	+	-	+	+	+	+
Cho et al., 2011	X	-	+	+	-	+	-	+
Chun and Yi, 2011	X	-	+	-	+	+	+	+
Eaton et al., 2022	-	+	+	+	+	+	+	+
Jiang and Wang, 2020	X	-	+	-	+	-	+	-
Kumar et al., 2018	X	-	+	+	+	-	+	-
Nasi et al., 2018	-	+	+	+	+	+	+	+
Piedra et al., 2014	-	+	+	+	-	+	-	+
Schuss et al., 2012	-	+	+	+	+	+	+	+
Sethi et al., 2022	X	-	+	+	+	-	+	-
Song et al., 2014	X	-	+	+	+	-	+	-
Songara et al., 2016	X	+	+	+	+	-	+	-
Tora et al., 2021	-	+	+	+	+	+	+	+
Vreeburg et al., 2024	+	+	+	+	+	+	+	+
Vyas and Singh, 2021	X	-	+	+	+	-	+	-
Yang et al., 2018	-	+	+	+	+	+	+	+
Zhang et al., 2010	X	-	+	+	+	-	+	-
Zhu et al., 2018	-	+	+	+	+	-	+	-

Risk of bias domains

Domains:
D1: Bias due to confounding.
D2: Bias due to selection of participants.
D3: Bias in classification of interventions.
D4: Bias due to deviations from intended interventions.
D5: Bias due to missing data.
D6: Bias in measurement of outcomes.
D7: Bias in selection of the reported result.

Judgement
X Serious
- Moderate
+ Low

Supplementary Table 3: Risk of bias summary for non-randomised studies (ROBINS-I).

Author	Critical Flaws	Non-critical Flaws	Overall confidence
Zheng et al., 2018	**Item 2** - No explicit protocol registered before conducting the review.<br>**Item 9** - Risk of bias assessment inappropriate: used Newcastle-Ottawa Scale (NOS), which AMSTAR 2 considers insufficient for detailed bias assessment of non-randomised studies.<br>**Item 15** - No assessment of publication bias.	**Item 5** - Only partial duplicate study selection and extraction (no clear statement that both phases were fully independent).<br>**Item 10** - Funding sources of included studies were not reported.<br>**Item 15** - Limited discussion of heterogeneity causes and potential impact on conclusions.	**Critically low**
Malcolm et al., 2018	**Item 2** - No explicit protocol registered before conducting the review.<br>**Item 9** - Risk of bias assessment inappropriate: used Newcastle-Ottawa Scale (NOS).<br>**Item 15** - No funnel plots, Egger's test, or discussion of small-study effects.	**Item 10** - Funding sources of included studies were not reported.<br>**Item 12** - Did not fully explore sources of heterogeneity across included studies.	**Critically low**
De Cola et al., 2018	**Item 2** - No protocol registration or published pre-specified methods. reproducible strategy reported.<br>**Item 9** - No formal risk of bias assessment; unclear how study quality influenced synthesis.<br>**Item 13** - Results discussed without integrating study-level biases into interpretation.	**Item 3** - Did not clearly justify inclusion/exclusion of different study designs.<br>**Item 10** - No information on primary study funding.<br>**Item 12** - Did not fully explore sources of heterogeneity across included studies.<br>**Item 16** - Conflicts of interest not explicitly stated.	**Critically low**
Palavani et al., 2025	**Item 2** - No prospective registration.<br>**Item 9** - Used MINORS scale but did not assess domain-specific biases (e.g. confounding, selection bias, reporting bias) as per ROBINS-I or Cochrane RoB.<br>**Item 15** - No funnel plots, Egger's test, or discussion of small-study effects.	**Item 10** - Funding sources for included studies not reported.<br>**Item 14** - Reported I^2 values but did not explore sources of heterogeneity.	**Critically low**
Chasles et al., 2025	**Item 9** - Risk of bias assessment inappropriate: used Newcastle-Ottawa Scale (NOS).<br>**Item 13** - Risk of bias scores not integrated into interpretation of findings.	**Item 10** - Funding sources for included studies not reported.<br>**Item 15** - Funnel plots visually inspected but no statistical tests.	**Critically low**
Thamilmaran & Patel et al., 2025 – this review.	**0**	**0**	**High**

Supplementary Table 5: Summary of Quality Appraisal of this study compared to previous systematic reviews using AMSTAR-2 criteria.

Early cranioplasty compared to Late cranioplasty for Traumatic brain injury

Certainty assessment							Summary of findings				
Participants (studies) Follow-up	Risk of bias	Inconsistency	Indirectness	Imprecision	Publication bias	Overall certainty of evidence	Study event rates (%)		Relative effect (95% CI)	Anticipated absolute effects	
							With Late cranioplasty	With Early cranioplasty		Risk with Late cranioplasty	Risk difference with Early cranioplasty
**Overall complications (assessed with: n)**											
1099 (8 non-randomised studies)	serious [a]	not serious	not serious	not serious	none	⨁⨁⨁◯ Moderate [a]	120/715 (16.8%)	76/384 (19.8%)	**RR 1.14** (0.82 to 1.58)	120/715 (16.8%)	**23 more per 1,000** (from 30 fewer to 97 more)
**Reoperation (assessed with: n)**											
975 (8 non-randomised studies)	serious [a]	not serious	not serious	not serious	none	⨁⨁⨁◯ Moderate [a]	23/597 (3.9%)	26/378 (6.9%)	**RR 1.75** (0.64 to 4.81)	23/597 (3.9%)	**29 more per 1,000** (from 14 fewer to 147 more)
**Functional outcome**											
391 (4 non-randomised studies)	serious [a]	not serious	not serious	not serious	none	⨁⨁⨁◯ Moderate [a]	272	119	-	272	MD **0.13 SD higher** (0.28 lower to 0.55 higher)

CI: confidence interval; **MD:** mean difference; **RR:** risk ratio

Explanations

a. Of the 18 studies, 10 were judged low risk of bias and 8 moderate risk

Supplementary Table 4: GRADE analysis.
